# Perianesthetic Complications in Genetic Mitochondrial Disease: A Review of Case Reports

**DOI:** 10.1002/pan.70257

**Published:** 2026-07-07

**Authors:** Brittany M. Johnson, Simon C. Johnson

**Affiliations:** ^1^ Translational Bioscience, Geography and Natural Sciences, Northumbria University Newcastle UK

**Keywords:** anesthesia, anesthetic complications, anesthetic outcomes, anesthetic toxicity, mitochondrial diseases

## Abstract

**Background:**

Genetic mitochondrial diseases (GMDs) are a large group of genetically and clinically heterogeneous disorders caused by defects in genes encoding mitochondrial components. GMDs are grouped into named syndromes based on clinical presentation, for example, Leigh syndrome (LS). Surgical interventions are often required in GMDs, necessitating the use of general anesthesia (GA). Some GMD animal models show both a marked reduction in anesthetic concentrations necessary for sedation and toxic sequelae resulting from anesthetic exposures. Hypersensitivity to sedation by anesthetic agents, most notably volatile anesthetic agents (VAs), occurs in a subset of GMD patients, and some evidence suggests toxicities are also present. Reported complications of anesthesia in GMD are varied, including minor metabolic changes, acceleration of underlying disease, and death. The potential toxicity of anesthetics in the setting of GMDs has recently been underscored by deaths among pediatric and young adult patients of Venezuelan descent putatively linked to a specific mitochondrial DNA haplotype. Interpretation of clinical findings is limited by the lack of a thorough review of case reports for anesthetic exposures in GMD patients. Here, we provide a comprehensive review of published case reports for GMD patients undergoing anesthetic exposures.

**Methods:**

Using search terms “anesthesia mitochondrial disease,” “anesthesia mitochondrial disease case report,” and “anesthesia Leigh syndrome” we identified case reports published up to May 2025. Non‐English articles were translated and included where possible. Only reports of GMD patients undergoing GA with total intravenous anesthesia (TIVA) or VAs containing outcome information were included, totaling 148 cases from 123 reports. We examined relationships between complications and GA type, clinical diagnosis, perianesthetic drugs, procedure length and type, and general demographics. We performed a narrative review of clinical and pre‐clinical literature.

**Results:**

Overall complication rate was significantly higher in VA versus TIVA cases (42% vs. 18%, Fisher's exact *p*‐value **p = 0.0022), as was rate of severe complications (*p = 0.01). Encephalomyelopathies, including LS, were overrepresented among cases resulting in death. Complication rate has remained relatively stable over time. Sex and age were not associated with significant differences in complication rate. Complication rate varied significantly by procedure type, and severe complications were associated with procedure length. While not statistically significant, older VAs had higher complication rates than newer VAs. Similarly, older neuromuscular blockade agents may be less safe than newer drugs. N_2_O and ketamine were notably safe among drugs used in TIVA procedures.

**Conclusions:**

Available case‐report data support the notion that GMD patients are at increased risk of a variety of perianesthetic complications. Clinical symptoms and procedure length appear most strongly predictive of severe complications. Clinical and pre‐clinical findings suggest more research is needed to understand the mechanisms of toxicity and more detailed reporting is needed in clinical case reports to identify potential risk factors.

## 1 Introduction

The umbrella term genetic mitochondrial disease (GMD) encompasses a large group of genetically and clinically heterogeneous diseases which are all caused by defects in genes encoding mitochondrial components. Individual GMDs can be ultrarare, but as a group they impact around 1 in 4000 individuals, representing a significant challenge for human health [1].

GMDs are highly variable in both genetic cause and clinical presentation. Heritability includes maternal inheritance in individuals with mitochondrial DNA (mtDNA) mutations; X‐linked heritability; autosomal recessive and autosomal dominant mutations; and complex heterozygosity. Over 400 unique genetic alterations have been linked to clinical GMDs [2]. In recent years exome and whole‐genome sequencing techniques have significantly enhanced detection of variants and genetic diagnosis, although validating the molecular impact and pathogenicity of novel putative causal variants lags. Clinical diagnosis still relies heavily on overall clinical features and biochemical study of mitochondrial respiratory function in skeletal muscle biopsy samples.

Symptoms in GMDs can include seizures, developmental delay, vision loss, neuroinflammatory brain lesions, musculoskeletal disease, metabolic dysfunction, etc. Many patients are born healthy, with no signs of disease at birth or early in life. Individual syndromes vary widely from single organ involvement, as in Leber's Hereditary Optic Neuropathy (LHON), to severe multisystem disorders, as in Leigh syndrome (LS) and Mitochondrial Encephalomyopathy, Lactic Acidosis, and Stroke‐like episodes (MELAS syndrome). Genetic heterogeneity is present in named syndromes; for example, over 110 genes are causally linked to LS [3, 4]. There is significant overlap between named GMDs in both clinical presentation and genetic cause.

Surgical interventions are often required in GMD patients, necessitating the use of general anesthesia with total intravenous (TIVA) or volatile anesthetic agents (VA). Profound hypersensitivity to anesthesia, specifically volatile anesthetic agents such as isoflurane and sevoflurane, is a clinical feature of some forms of GMD [5]. This sensitivity is most strongly linked to defects in mitochondrial electron transport chain complex I (ETC CI, NADH–ubiquinone oxidoreductase). Animal models of GMD, including the *Ndufs4*(−/−) mouse model of LS, similarly show hypersensitivity to anesthesia with VAs and propofol [6].

In addition to sensitivity, GA in animal models has been linked to toxic perianesthetic sequelae including metabolic disruption, respiratory failure, poor recovery from anesthesia, and death [7, 8, 9]. In the *Ndufs4(−/−) model*, brief exposure to isoflurane at low concentrations resulted in some perianesthetic deaths and an acceleration of disease progression in surviving animals. Case reports support the notion that human GMD patients are also susceptible to anesthetic toxicities and clinical complications. These vary widely and include minor metabolic changes, acceleration of neurodegenerative disease, and even death. The potential toxicity of anesthetics in the setting of GMDs has recently been underscored by perianesthetic deaths among pediatric patients of Venezuelan descent who appear to carry mutations in the mtDNA encoded gene *MT‐ND4*, a subunit of ETC CI, that impacts VA responses [10, 11]. Notably, it seems that none of these patients had any signs of disease prior to anesthetic exposure.

While available clinical reports reveal various perianaesthetic complications associated with GA in GMD patients, interpretation of clinical findings and the development of recommendations are limited by the lack of a thorough review of relevant case reports. Here, we provide a comprehensive overview of published case reports for GMD patients undergoing anesthetic exposures, emphasizing types of procedures and reported complications, and a brief discussion of anesthesia toxicities in animal models.

## 2 Methods

### 2.1 Case Report Identification and Inclusion Criteria

**FIGURE 1:**
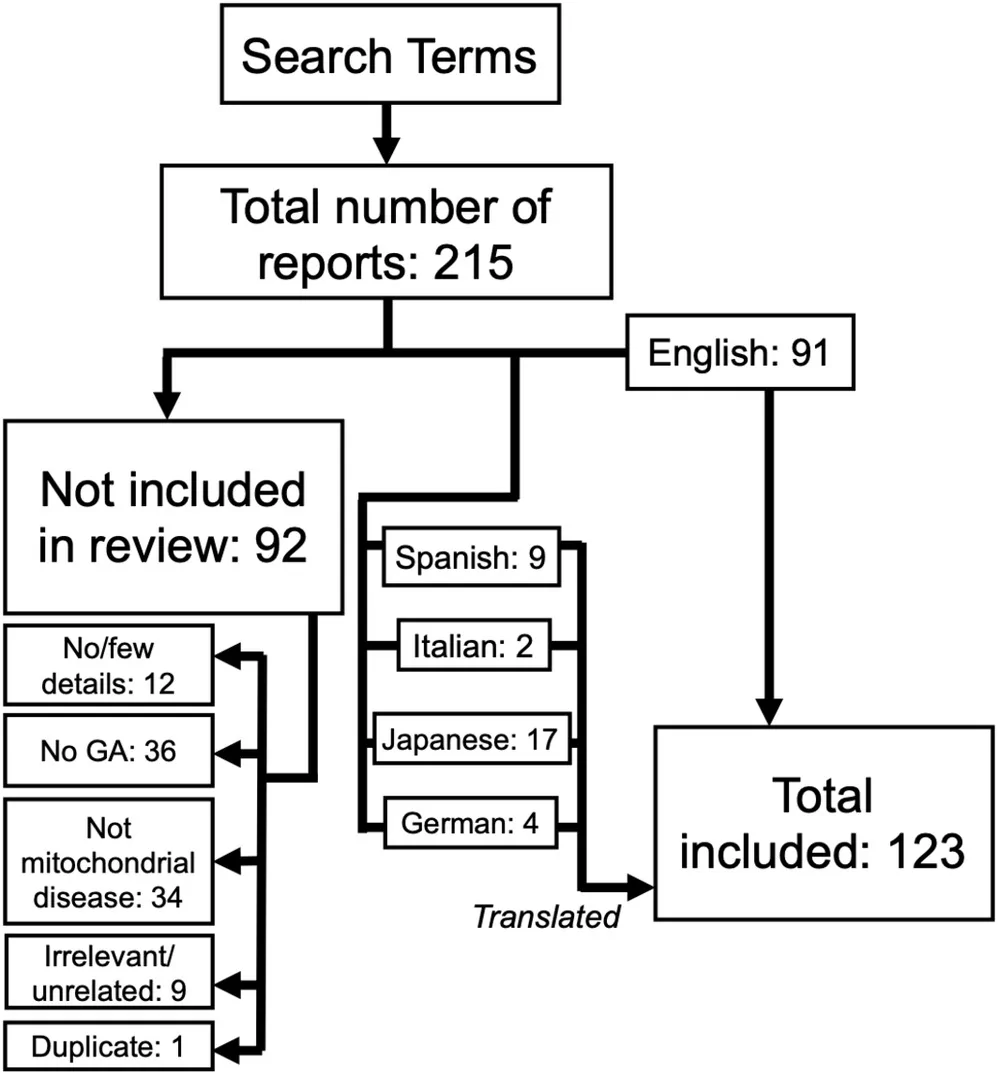
Review of case reports on anesthesia in GMD. Seach terms “anesthesia mitochondrial disease, “anesthesia mitochondrial disease case report,” and “anesthesia Leigh syndrome,” with spelling variations, were used to query PubMed. All resulting manuscripts up until May 2025 were considered, with inclusion or exclusion as detailed in this flow chart and in Section 2. Non‐English articles were translated and included. Only reports of GMD patients undergoing GA with TIVA or VAs (not local anesthetics only), and only cases with outcome information, were included, yielding 148 total cases from 123 case reports. Among those manuscripts not included were 76 in English, 7 in German, 4 in Japanese, 4 in Spanish, 1 in French. A summary of all manuscripts included is provided in Supporting Information.

PubMed was searched using the terms “anesthesia mitochondrial disease,” “anesthesia mitochondrial disease case report,” and “anesthesia Leigh syndrome” and all resulting manuscripts were considered, with a date range of January 1, 1935 up until May 31, 2025. Additional variants of these search terms did not yield additional relevant studies. Non‐English articles were translated and included where possible. Only reports of GMD patients undergoing GA with TIVA or VAs (not local anesthetics only), and only cases with outcome information, were included, yielding 148 total cases from 123 case reports (Figure 1). Relevant assessed details for all these manuscripts are provided in Supporting Information.

### 2.2 Assessing Complication Rates

Our primary goal in this review is to examine complication rates in case reports for GMD patients undergoing anesthetic procedures. Accordingly, we first established criteria for defining complications and severity. All cases were assigned the designation of uncomplicated or were assigned a complication score of 1, 2, or 3, depending on severity. Category 1 were defined as those considered mild. These included metabolic changes that resolved quickly, For example, increased lactate, increased liver/renal values, hypercapnia, metabolic acidosis/alkalosis. Category 2 were defined as those considered moderate and included potentially life‐threatening physiologic disturbances such as hypothermia, pyrexia, bradycardia, bradypnea, and sustained metabolic changes such as metabolic acidosis. Category 3, defined as severe, included overtly life‐threatening changes such as cardiovascular collapse, marked arrhythmia, respiratory failure, sepsis, marked metabolic changes that resulted in decompensation, and perianaesthetic death. No patient in these case reports died during anesthesia, deaths occurring beyond the perianesthetic period were included only if it was deemed likely that GA played a proximal role. Many additional deaths were reported to occur in the months after GA procedures, but available data are insufficient to explore any role for GA in accelerating disease progression or death (see Section 4).

### 2.3 Statistical and Descriptive Analyses

We examined relationships between complications and GA type; clinical diagnosis; perianesthetic drugs used; procedure length and type; and general demographics. The assessed details for all manuscripts included in this review are provided in Supporting Information. The type, quality, genetic and clinical heterogeneity, and sometimes limited information content of available case report studies limited applicable statistical approaches to the data (see Section 4). Specifically, robust multivariate analysis of outcomes is not possible. Instead, we employ descriptive and inferential statistics to examine overall trends in the available data, with caveats noted in our discussion. These include *t*‐tests and ANOVA for differences between groups, and Fisher's exact tests for differences in categorical data. All statistical tests were performed using GraphPad Prism and are detailed in relevant table and figure legends.

## 3 Results

### 3.1 Complication Rates Over Time

**FIGURE 2:**
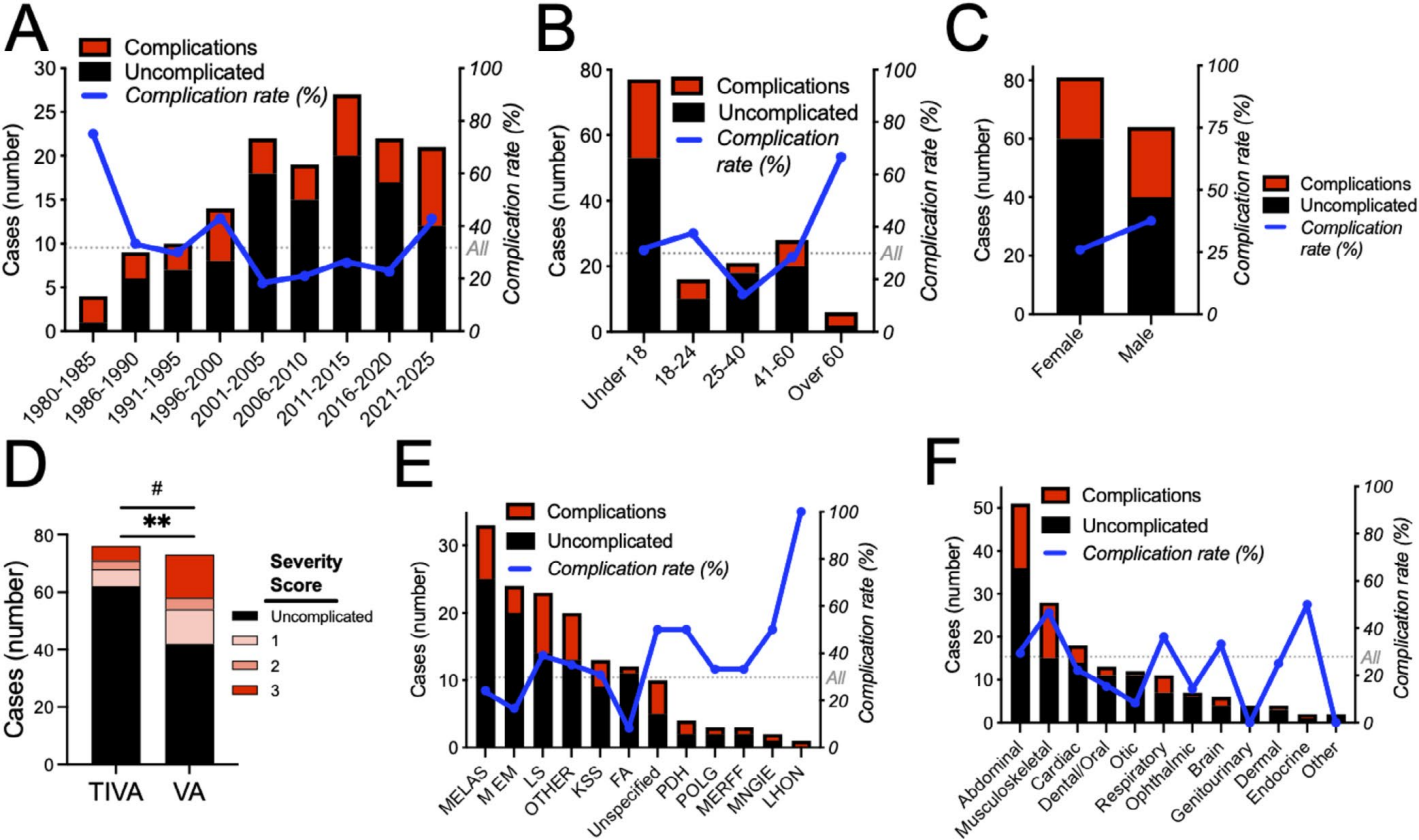
Complications among anesthesia procedures in patients with genetic mitochondrial disease—demographics and total intravenous anesthesia versus volatile anesthetics. (A) Number of uncomplicated and complicated case reports (black and red stacked bars, respectively) and complication rate (blue line) as a function of time in 5‐year bins. (B) Number of uncomplicated and complicated case reports (black and red stacked bars, respectively) and complication rate (blue line) as a function of patient age. (C) Number of uncomplicated and complicated case reports (black and red stacked bars, respectively) and complication rate (blue line) in males and females. (D) Number of uncomplicated and complicated cases, split into severity group (see Section 2) in total intravenous anesthesia (TIVA) and volatile anesthetic (VA) cases. Total and severe complications are significantly higher among VA vs. TIVA cases. Fisher's exact test **p = 0.002 for complication rate (total); Fisher's exact test #*p* = 0.01 for severe complications. (E) Number of uncomplicated and complicated case reports (black and red stacked bars, respectively) and complication rate (blue line) by clinical diagnosis (see Table S1). (F) Number of uncomplicated and complicated case reports (black and red stacked bars, respectively) and complication rate (blue line) by procedure type (see Section 2, Table S2).

First, to assess whether the frequency of complications has changed over time, we examined overall complication rate over time by pooling all severity categories (see Section 2) and grouping reports into 5‐year bins starting with 1980–1985, when the first relevant case‐reports appear (Figure 2A). The number of case reports detailing GA in GMD patients increases by over five‐fold from 4 in the 1980–1985 bin to 21 in the 2001–2005 bin and subsequently remained at 15–20 (~3–5 per year). After the first bin, the frequency of perianesthetic complications is relatively stable, fluctuating between ~15%–20%, with upticks to ~40% in the 1996–2000 and most recent 2021–2025 bins.

### 3.2 Complications by General Demographics—Age and Sex

Complications were reported in patients ranging in age from 4 weeks to 69 years (see Supporting Information). When comparing age groups by pediatric (0–18), young adult (18–24), adult (25–40), older adult (41–60), and senior (over 60), the greatest number of cases occurs in the pediatric group (Figure 2B). Overall complication rate is similar across age groups; those over 60 show the highest rate (66%), but the sample size in this group is small (6 individuals).

Although female cases are more prevalent (81 versus 64, respectively), males have a higher frequency of complications (37.5% vs. 26%, see Figure 2C).

### 3.3 TIVA Vs. VA

Procedures were split into two groups based on overall anesthetic approach—TIVA (total intravenous anesthesia) and VA (general anesthesia maintained with a volatile anesthetic). Complications occurred in 14 of 76 TIVA cases (~18%) compared to 31 of 72 VA cases (~43%), an approximately 2‐fold higher rate in VA procedures (Fisher's exact *p*‐value **p = 0.0022, Figure 2D). TIVA and VA cases also significantly differ in the rate of severe category complications (20.5% among VA vs. 6.6% in TIVA, Fisher's exact *p*‐value #*p* = 0.01). The number of deaths likely to be caused at least in part by the GA is 3 and 2 for VA and TIVA cases, respectively (see Table 1; for a narrative summary of each category 3 case see Supporting Information Category 3 Cases).

**TABLE 1:** Overview of complications. A summary of complications detailed in case reports of anesthetic procedures in GMD patients.

TIVA cases (14 cases)						
Disease	Procedure type	Complication(s)	Severity score (1–3)	Duration (min)	Year	Citation
MNGIE	Abdominal	Pain	1	110	2021	[12]
Mitochondrial myopathy	Abdominal	NMB effect persisted 45–60 min, suspect sensitivity	1	130	1998	[13]
KSS	Cardiac	Increased lactate	1	100	2023	[14]
KSS	Brain	Increased LDH, GOT, GPT	1	220	1993	[15]
MELAS	Abdominal	Mild hypercarbia, hypothermia, hyponatremia	1	140	2009	[16]
MELAS	Cardiac	Metabolic acidosis	1	264	2022	[17]
ETC CI deficiency	Musculoskeletal	Pyrexia 48 h	2	150	1998	[18]
MELAS	Abdominal	Hypothermia	2	150	2003	[19]
Unspecified	Abdominal	Weak, slow breathing	2	45	2021	[20]
Leigh	Musculoskeletal	Pyrexia, inspiratory stridor, hyperlactatemia, hyperpyruvatemia, obtundation.	3	Not reported	1990	[21]
Mito encephalopathy	Respiratory	Profound hypotension, sinus bradycardia, ventricular tachycardia, cardiac arrest	3	Not reported	2013	[22]
Unspecified	Oral	Hyperglycemia, transient supraventricular tachycardia, brief sinus block, apnea	3	160	2017	[23]
Unspecified	Abdominal	Severe respiratory depression, septicemia. * Death after 6 weeks	3	480	2004	[24]
Unspecified	Musculoskeletal	Metabolic acidosis, hyperlactatemia, sinus bradycardia. * Death after 2 weeks	3	Not reported	2013	[25]

VA Cases (31 cases)						
Disease	Procedure type	Complication(s)	Severity score (1–3)	Duration (min)	Year	PMID
PDH Deficiency	Musculoskeletal	Hyperlactatemia	1	150	2008	[26]
CPEO	Abdominal	Postoperative mild hyperkalemia	1	NR	2016	[27]
MERFF	Dermal	Hypothermia	1	170	2021	[28]
Complex I deficiency	Abdominal	Prolonged sedation	1	48	2024	[29]
Glutaric aciduria type 1	Musculoskeletal	Vomiting	1	45	2011	[30]
Leigh	Musculoskeletal	Low PT	1	300	2004	[31]
Leigh	Ophthalmic	Pyrexia (38.1C)	1	169	1996	[32]
Unspecified mito; likely Leigh	Abdominal	Hyperglycemia, hyperlactatemia	1	NR	1997	[33]
MELAS	Abdominal	Metabolic acidosis	1	160	2016	[34]
MELAS	Musculoskeletal	Hyperlactatemia	1	70	2011	[35]
MELAS	Abdominal	Hyperglycemia, metabolic alkalosis	1	210	2011	[35]
MELAS	Abdominal	Hyperglycemia	1	175	2011	[35]
KSS	Respiratory	Sinus bradycardia	2	Not reported	1994	[36]
POLG	Abdominal	Moderate hypotension	2	167	2022	[37]
Congenital proprionic acidemia	Cardiac	Excessive blood loss	2	Not reported	2024	[38]
Leigh	Otic/Ophthalmic	Hyperlactatemia, hyperpyruvatemia, severe developmental regression, cerebral degenerative disease (CT scan)	2	Not reported	1981	[39]
KSS	Abdominal	Dyspnea, cyanosis, respiratory depression, ventricular tachycardia	3	55	1995	[40]
PDH Deficiency	Abdominal	Base deficit, hyperlactatemia, septicemia, respiratory distress, seizures. * Death after 30 days	3	Not reported	1983	[41]
MELAS	Endocrine	Upper airway narrowing with bilateral recurrent laryngeal nerve paralysis	3	Not reported	2016	[27]
Mito depletion	Abdominal	Hypotension, metabolic acidosis, hyperlactatemia, hyperglycemia, post‐reperfusion syndrome	3	605	2024	[42]
AMA positive myositis	Musculoskeletal	Severe bradycardia, supraventricular tachycardia, sinus arrest	3	Not reported	2023	[43]
FA	Musculoskeletal	Supraventricular tachycardia, hypertension, hypothermia, marked metabolic acidosis	3	240	1984	[44]
MELAS	Cardiac	Adrenal insufficiency, acute renal failure	3	540	2011	[35]
MELAS	Musculoskeletal	Ventricular tachycardia, hypotension, septicemia, hyperglycemia	3	247	2011	[35]
Leigh	Musculoskeletal	Clinical deterioration with pulmonary consolidation, suspected septicemia, apnea, cerebral and ventricular changes with new progressive necrosis	3	420	2003	[45]
Leigh	Musculoskeletal	Marked metabolic acidosis and respiratory acidosis, hyperglycemia, tachycardic	3	Not reported	1996	[32]
Leigh	Brain	Apnea, hypertension, seizure. * Death 34 h after procedure	3	Not reported	1990	[21]
Leigh	Respiratory	Apnea, hypotonia, stridor, hyperlactatemia, hyperpyruvatemia, brain lesions (CT scan). * Death 5 days after procedure	3	Not reported	1990	[21]
Leigh/LHON	Otic	Clinical deterioration, ataxia, opsoclonus, myoclonus, language and motor regression	3	Not reported	2019	[46]
Unspecified mito encephalopathy	Abdominal	Respiratory depression, hypotension	3	Not reported	2009	[47]
Unspecified mito encephalopathy	Dental	Apnea, respiratory depression	3	Not reported	2006	[48]

Abbreviations: AMA, Anti‐mitochondrial antibody; CPEO, Chronic external ophthalmoplegia; CT, Computed tomography; ETC CI, Mitochondrial electron transport chain complex I; FA, Friedrich's Ataxia; GOT, Glutamate oxaloacetate transaminase; GPT, Glutamate pyruvic transaminase; KSS, Kearns‐Sayre Syndrome; KSS, Kearns‐Sayre Syndrome; LDH, Lactate dehydrogenase; LHON, Leber's hereditary optic neuropathy; MELAS, Mitochondrial encephalopathy, lactic acidosis, and stroke‐like episodes; MERFF, Myoclonic epilepsy with ragged red fibers; MNGIE, Mitochondrial neurogastrointestinal encephalopathy syndrome; NMB, Neuromuscular blockade; PDH, Pyruvate dehydrogenase; POLG, DNA polymerase subunit enzyme gamma deficiency; PT, Prothrombin time; TIVA, Total intravenous anesthesia; VA, General anesthesia with volatile anesthetics.

### 3.4 Complication Rates by Clinical Diagnosis

While they share some aspects of molecular etiology, individual syndromes included in the GMD umbrella are diverse in specific genetic cause as well as clinical features (see Section 1 and Section 4). We next assessed complication rate by clinical diagnosis, For example, Mitochondrial Encephalomyopathy, Lactic Acidosis, and Stroke‐like episodes (MELAS), Leigh syndrome (LS), etc. (Table S1, Figure 2E). Among those with 10 or more cases, complications are most frequent in patients with unspecified mitochondrial disease (mitochondrial dysfunction with no named syndrome) at 50%, followed by LS at ~39%. Friedreich's ataxia (FA) patients have the lowest rate of complications at ~8% in that group.

Those two TIVA patients who died were both unspecified mitochondrial disease patients and suffered from metabolic disruptions, including hyperlactatemia.

In VA cases, metabolic disruptions are a recurring complication among MELAS patients (Table 1). Respiratory issues (apnea, respiratory depression, etc.) and metabolic disruption are common among LS and unspecified mitochondrial encephalopathy patients (see Section 4).

Among VA procedures, patients diagnosed with LS account for 12 of 73 total cases (~16%) but represent 5 of 15 (33.3%) category 3 (severe) complications (see Table 1). The remaining severe complications cases include two unspecified mitochondrial encephalopathies and a PDH deficiency, which share pathophysiology (former) and genetic cause (latter) with LS (see Section 4). Two of the three deaths reported in VA cases were LS patients, the third a PDH deficiency case (which overlaps with LS).

### 3.5 Complications by Procedure Type

To assess whether there were any obvious links between procedure type and complications we assigned procedures to categories based on the organ system involved: abdominal, cardiac, brain, musculoskeletal, respiratory, oral, ophthalmic, otic, endocrine, and dermal (see Supporting Information for the full list of exact procedures). Procedure types vary in all clinical diagnoses (see Table S2). Abdominal cases are the most common in MELAS patients, while musculoskeletal cases are the most common in FA and LS patients. Complication rates appear higher than average for musculoskeletal procedures and lower than average for otic, ophthalmic, and genitourinary procedures. However, no comparison to the total complication rate is statistically significant (Figure 2F).

### 3.6 Procedures in GMDs

Surgical interventions are common in some forms of GMDs. Among published case reports, reasons for surgical anesthesia varied widely by clinical diagnosis (see Table S2). For example, cardiac procedures were most common among Kearns‐Sayre syndrome (KSS) patients, while abdominal procedures made up the majority of MELAS patient cases. Musculoskeletal procedures were the most common in FA patients and LS patients. Notably, this procedure category had the highest rate of complications overall, but complications were reported in a disease‐specific manner—FA patients show the lowest complication rate overall, while LS patients were among the highest (Table S1). Accordingly, enrichment of a certain procedure category in a particular GMD cannot account for rates of anesthetic complications in that disease.

**FIGURE 3:**
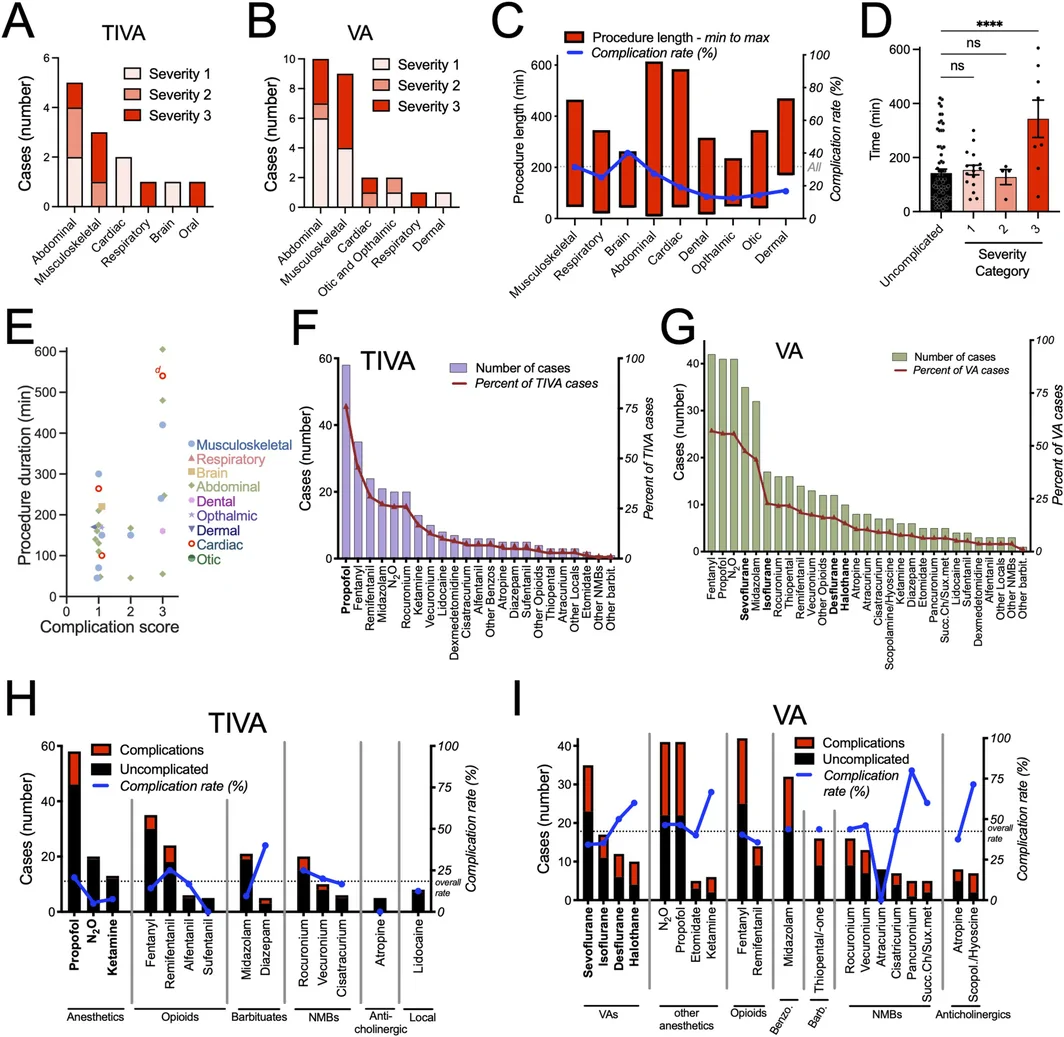
Complications in detail. (A) Complication numbers, by severity, among procedure types in TIVA cases. (B) Complication numbers, by severity, among procedure types in VA cases. (C) Procedure length (min to max, red bar) and complication rate (blue line) (see Table S3). (D) Procedure length by severity score. Severity 3 cases were significantly longer than uncomplicated cases—ANOVA ***p < 0.0003, Dunnet's multiple testing corrected pairwise *t*‐test ****p < 0.0001. (E) Procedure duration by complication with procedure type annotated. Lower case red *d* denotes the one death where the length of the procedure was reported. (F) Pharmacologic agents reported in TIVA cases—number of individual TIVA cases where the drug was used (bar) and percent of TIVA reports (red line). See Table S4. (G) Pharmacologic agents reported in VA cases—number of individual VA cases where the drug was used (bar) and percent of VA reports (red line). Table S5. (H) Uncomplicated and complicated cases (black and red stacked bars) by drug and complication rate (blue line) for TIVA cases. Only those with 5 or more total cases plotted. Anesthetic agents are bolded, each case in TIVA included one or more of these. (I) Uncomplicated and complicated cases (black and red stacked bars) by drug and complication rate (blue line) for TIVA cases. Only those with 5 or more total cases plotted. Volatile anesthetics are bolded, each case in VA included one of these.

Given the noted difference in complication rate in TIVA vs. VA procedures, we next considered these groups separately. As with the total case number in the combined data (Figure 2D), anesthetic complications are most abundant in abdominal, musculoskeletal, and cardiac cases, in that order, in both TIVA and VA anesthesia (Figure 3A,B, Table 1). Musculoskeletal cases make up the largest group of the most severe (severity 3, see Section 2) complications for both TIVA and VA anesthesia cases, further suggesting musculoskeletal cases may represent the highest risk as suggested by the overall frequency data.

Among deaths, two are TIVA cases, while three are VA cases (Table 1). Abdominal and musculoskeletal procedures accounted for deaths in TIVA cases, while abdominal, respiratory, and brain procedures account for deaths in VA cases, showing notable variation in the type of procedure associated with perianesthetic death.

### 3.7 Duration

Procedure length varies widely in both TIVA and VA anesthesia cases (Figure 3C). Complication rate appears higher than expected in brain cases given the procedure time, while dermal, dental, otic, and ophthalmic procedures appear to have lower than expected complications given their length, but statistical comparisons are limited by low case numbers (see Figure 3C, Table S3).

When factoring in complication severity, procedure time is not significantly higher in severity category 1 or 2 groups compared to uncomplicated cases. However, average duration is significantly higher in the category 3 complication group compared to uncomplicated cases (Figure 3D). Plotting severity by duration indicates the relationship between procedure length and severity applies to multiple procedure types (Figure 3E). Notably, the one death where the duration was reported is the third longest of all procedure lengths available (marked with a red *d* in Figure 3E). Procedure length was not reported in the other cases where death occurred (see Table 1).

### 3.8 Drugs Used During GMD Anesthesia Management

A variety of anesthetic agents and other drugs are used in the anesthetic management of GMD patients. Reported anesthetic agents include propofol, N_2_O, sevoflurane, isoflurane, halothane, desflurane, ketamine, and etomidate. Additional drugs include analgesics such as fentanyl and other opioids; neuromuscular blockade (NMB) agents including succinylcholine; anticholinergics such as atropine; and benzodiazepines such as midazolam (Tables S4 and S5, Figure 3F–I). A few patients were also given agents for medical reasons other than anesthesia (antihistamines, GI protectants, anti‐seizure medication, etc.).

In both TIVA and VA, propofol is a commonly used agent, at ~75% and 54%, respectively (Figure 3G). Fentanyl is also commonly used (~45% and 55% in TIVA and VA procedures). N_2_O and the benzodiazepine midazolam are common among VA procedures.

In both TIVA and VA, fentanyl is the most frequently administered opioid, while midazolam was the most commonly used benzodiazepine. Rocuronium and vecuronium are the most common neuromuscular blocking agents. Accordingly, the overall landscape of adjunct pharmacologic agents appears very similar between TIVA and VA procedures.

Among VAs employed in VA procedures, sevoflurane is the most frequently reported, followed by isoflurane, desflurane, and halothane (see Figure 3F–I, see Section 4). Propofol and N_2_O are commonly reported induction agents for VA procedures (see Table S5), while N_2_O is common during maintenance.

To ascertain whether any individual agent appears to be associated with increased complication rates, we compared complication rates for each agent against the background rate by general anesthetic approach (TIVA or VA). Some trends of potential interest were present—for example, desflurane and halothane appear to be associated with higher rates of complications than isoflurane and sevoflurane in VA cases, and N_2_O and ketamine appear to be particularly safe agents in TIVA cases (see Section 4). However, low numbers of individual studies limit the statistical power of comparisons.

## 4 Discussion

### 4.1 Key Findings and Clinical Relevance

Though limited by the quantity, quality, and detail level of available case reports, the review here provides a useful overview of the clinical landscape of anesthetic procedures and complications associated with them in GMD, and identifies some interesting relationships.

A few observations stand out. Foremost, available evidence indicates that complications associated with GA are a continued concern in the management of patients with GMD. Notably, the number and fraction of cases with complications have remained relatively stable over time. Reported complications range from mild transient physiologic disturbances to severe metabolic, respiratory, and cardiovascular derangements, as well as death. A great deal remains unknown regarding specific risk factors, and further study is clearly warranted.

Beyond this broad conclusion, we can report some specific findings. For example, while complications occur in both TIVA and VA anesthetic procedures, VA procedures are associated with a statistically greater number of total and severe (Category 3) complications (see *Caveats* below). While supportive animal model data is extremely limited, this is consistent with the greater sensitivity to isoflurane than propofol in the mouse model of LS [6]. Isoflurane has been linked to toxic sequelae in this model, but possible toxicities of propofol have not been similarly explored [8, 9].

We also find, perhaps not surprisingly, that average procedure length is significantly higher in those cases with severe complications compared to those with no complications. In contrast, procedure length does not vary significantly between uncomplicated and mild or moderate cases.

We additionally see that LS cases are enriched among VA cases with category 3 complications. LS complications typically include metabolic changes and respiratory dysfunction, consistent with data from the mouse model [7, 8, 9].

These observations provide tangible points of consideration for anesthesiologists developing guidelines for the care of GMD patients. Specifically, (1) it may be reasonable to explore additional monitoring for lengthy procedures, (2) TIVA appears to be a generally safer approach compared to VA anesthesia in GMD patients, and (3) a diagnosis of LS could reasonably lead to increased monitoring in any GA procedure, but particularly any procedure involving VAs. Qualitatively the summary of clinical data reveals that poor responses commonly included metabolic and respiratory disruptions, providing possible targets for increased monitoring (e.g., serial blood lactate monitoring, etc.).

For a narrative summary of all category 3 cases see Supporting Information Category 3 Cases.

### 4.2 Notable Trends in Specific Drugs

Though preliminary, comparisons of complication rates between drugs in the same class may provide insight into the relative safety of anesthetics and adjunct compounds. For example, among NMB agents, cases employing pancuronium have a higher than average rate of complications, while complications were lower than average in those using atracurium. While preliminary and lacking any mechanistic explanation, it is worth noting that in the general population pancuronium has been shown to have poor onset and recovery times compared to the newer NMB vecuronium [49].

Similar to atracurium, N_2_O and ketamine stand out as apparently safe compounds—rates of complications are particularly low in TIVA cases where these were used, indicating they alone cannot be key drivers of complication rate.

The available data also suggests newer VAs (sevoflurane and isoflurane) are associated with lower rates of complications compared to older, outdated VAs (halothane and desflurane). This comparison is possible as VA procedure patients are only provided one VA compound; furthermore, given that complication rates have remained relatively stable over time we can tentatively conclude the differential rate of complications in newer vs. older VAs is not due to unrelated changes to anesthetic procedures as a function of time.

While these trends are correlative (see *Caveats*), they suggest that compounds selected for greatest safety in the general population will generally also be the safest in GMD patients—a meaningful finding that may contribute to the development of recommendations for clinical management.

Notably, while increased risk of propofol infusion syndrome (PRIS) has been associated with GMDs, for example in the management of intractable epilepsy [50], in the literature reviewed here only the lengthy (88 h) multi‐trauma case reported PRIS as possibly contributing to perianesthetic complications. Accordingly, it is not clear from the available literature whether GMD patients are at higher risk of PRIS.

### 4.3 NMBs

NMB sensitivity and recovery may play an important role in respiratory complications, though the data available are too limited to form strong conclusions. At least three studies have reported increased sensitivity or prolonged recovery from NMBs in GMD patients, two with unspecified mitochondrial myopathies and one with a POLG‐related mitochondrial disease [13, 37, 51]. Among complications, four of six TIVA cases with reported respiratory complications used NMB agents, with two of the four using reversal agents. Four out of the eight VA cases with respiratory complications used NMB agents; none reported using reversals. Among respiratory procedures themselves, only one of the three reported using an NMB which was not reversed. The reversal agent neostigmine was used in 14 cases (four with complications), while sugammadex was used in two cases, one reporting complications. As others have noted, adequate monitoring and reversal of NMDs should be a consideration in developing clinical recommendations.

### 4.4 Insights From Animal Models of GMD

While a large body of evidence suggests that VAs are capable of disrupting mitochondrial function (e.g., [52, 53, 54, 55, 56]), few studies have directly examined anesthesia‐related outcomes in animal models of GMD.

Some data from invertebrate models suggests they may be useful in further dissecting mechanisms of anesthetic toxicity and identifying strategies for intervention. In Drosophila, mutations in the mitochondrial ETC CI component ND23 has been found to result in an oxygen‐dependent sensitivity to neurotoxicity mediated death as a result of isoflurane, but not sevoflurane, exposure [57]. The nematode *C. elegans* was, of course, the first model directly linking genetic defects in mitochondrial function to hypersensitivity to anesthesia [58]. *C. elegans* have been used to probe mechanisms of anesthesia‐induced neurotoxicity during development, suggesting they may provide a useful model for further study of anesthesia toxicity in the setting of GMD‐related defects [59].

In the *Ndufs4*(−/−) mouse model, isoflurane exposures accelerate mortality in a dose‐ and duration‐dependent manner. Notably, oxygen concentration contributed to some, but not all, of the observed toxic sequelae associated with isoflurane exposure; metabolic derangements such as hyperlactatemia were driven by isoflurane alone [8]. Isoflurane also dose‐dependently induced weight loss and accelerated disease progression in the model in the days following the exposure, regardless of oxygen concentration. In contrast, oxygen was the primary driver of perianesthetic mortality. Notably, toxic sequelae resulting from isoflurane exposures only occurred after disease onset and were prevented by the immune‐depleting drug pexidartinib, which prevents symptoms of LS in this model [7, 8].

While much of anesthesia sensitivity is undoubtedly driven by neuronal function and acute physiologic responses, the data from the mouse model suggests that some complications may be caused by other specific, targetable, pathophysiologic mechanisms. For example, increased blood lactate is a common GA complication in GMD patients often present in patients suffering severe complications including death (see Table 1), and metabolic derangements were among the sequelae prevented by immune depletion in the LS mouse model [7]. The mouse model also has the potential to identify contraindicated treatments. Dietary ketosis, for example, has been used to treat intractable seizures in GMD, and was found to unexpectedly exacerbate VA‐induced metabolic dysregulation in the LS mouse model [9]. Whether these observations are true in other GMDs, or can be leveraged to develop effective clinical interventions or reduce iatrogenic toxicities resulting from interactions between anesthesia and treatments, remains to be seen.

### 4.5 Acute Toxicities and the Need for Further Study

Recent reports of deaths in pediatric anesthesia patients of Venezuelan descent putatively driven by mutations in mitochondrial encoded *mt‐ND4* have raised awareness of the links between anesthesia and mitochondrial disease [10, 11]. Available information is limited, but it appears only one had neurologic symptoms prior to undergoing anesthesia, suggesting these patients would not have been recognized as GMD patients prior to GA, and even after GA may have limited or no signs of any underlying pathology. The considerations underlying clinical recommendations for at‐risk populations are complex and beyond the scope of this review, but these cases are worth highlighting for a few reasons. First, they may provide the first example of a form of mitochondrial dysfunction where anesthesia responses are the most prominent, or perhaps even only, clinical presentation. If so, whether they should be considered GMD patients is somewhat unclear. Other, subtler, symptoms may be present or arise later in life. Second, these cases provide clear examples of the reason GA needs to be carefully approached in suspected GMD patients, as many of the case reports detailed here state. Third, the Venezuelan cases demonstrate that carriers of genetic risk factors may not always present with clinical features of disease. Accordingly, family history should be considered in this context. Finally, the Venezuelan cases clearly support the need for more research on both the basic biology of anesthesia and the interactions between VAs and GA.

Unfortunately, while the topic of anesthesia induced neurotoxicity (AIN) in pediatric patients attracted massive attention [60, 61, 62, 63, 64], funders continue to respond to grants on the topic of anesthesia in GMD by stating it is an unimportant topic. It is possible that the negative results in the AIN field have led to skepticism about the potential for anesthesia toxicity more broadly. To this point, we note that our group published data highly skeptical of AIN findings in mice, but have shown robust toxicities in mouse models of GMD [61]. Data linking genetic defects impacting mitochondrial function to toxicity may suggest that the phenomena of AIN may exist in certain genetic backgrounds. We hope the attention given to the recent mitochondria‐associated GA deaths ultimately leads to increased proclivity to fund studies on these important topics.

### 4.6 Long‐Term Effects of GA

The data reviewed here are generally limited to acute, or acute on chronic, sequelae. Longitudinal studies have revealed that VA exposures accelerate underlying GMD progression in the mouse model, as noted above. Whether GA procedures in humans lead to neurotoxicity or long term acceleration of disease is unknown. Any anesthesia toxicities may be limited to specific clinical syndromes or genetic causes. The genetic and clinical heterogeneity of GMD patients greatly complicates study in humans; longitudinal datasets monitoring patients prior to and following GA procedures should help resolve some of these questions. Some questions, such as the relative risk of GA procedures in pre‐symptomatic vs. symptomatic patients, would require extremely large scale longitudinal studies of GA outcomes. In these cases, animal models may continue to provide some insight where human studies are limited.

### 4.7 Limitations, Caveats, and the Need for Improved Reporting

Here we have provided an overview of GA procedures and perianesthetic complications in GMD patients. Available case reports provide some key insights and reveal notable correlations worth further study, discussed below. However, the studies included and our synthesis of them suffer from limitations and caveats.

An obvious limitation here is the low number of overall reports, clearly representing only a small fraction of GMD patient GA procedures. While estimates of overall procedure frequency in GMD are lacking, a recent registry of 116 genetically confirmed LS patients reported that ~30% underwent tracheotomy procedures alone [3]. LS patients often undergo procedures for a variety of other reasons; the number of LS patients undergoing at least one GA procedure is likely much greater than 30%. Unfortunately, the limited reporting makes it difficult to assess specific sources of risk. For example, the current dataset was not sufficient to explore whether any specific genetic causes (or shared molecular targets of genetic defects) are linked to the risk of complications. A more alarming limitation was the frequent lack of information regarding the carrier gas used, in particular the oxygen concentration, precluding any analysis of whether the carrier gas impacts outcomes (as predicted by animal data, see above). High oxygen concentrations continue to be commonly used in pediatric anesthetic procedures, with a recent (2024) review of 226 cases at one children's hospital found that perianesthetic oxygen is barely regulated and ~90% of cases reviewed were hyperoxic by FiO_2_ [65]. Given the reproducible toxic effects of high oxygen concentration in GMD patients (and other settings), improvements in both the regulation and reporting of oxygen concentrations would appear urgent. A greater depth of coverage for case reports will lead to a better picture of anesthesia in GMD.

While the low coverage limits what questions can be asked, *relative* comparisons, where reporting biases are not thought to be present, can still provide insights. For example, we have no reason to think reporting biases might impact comparisons of procedure length and complication severity or the fraction of reported cases with complications over time. In contrast, we can draw no conclusions regarding actual total rates of complications, as cases with complications are probably more likely to be published than those which are uneventful. Here we only provide analyses we believe are not subject to reporting biases. However, we are limited by the case report literature available, and the observations reported here should be interpreted as hypothesis generating and preliminary, requiring further study.

A key issue with the case reports evaluated here is a lack of detailed reporting, as noted regarding carrier gas oxygen concentration. Many have incomplete or missing core details regarding the anesthetic and surgical procedures themselves such as procedure length, concentration of gas anesthetic used, carrier gas, etc. Adjunct drugs used are also often omitted, as is the carrier gas for volatile anesthetics. Future case reports and efforts to gain insights from clinical findings would benefit greatly from adopting basic reporting criteria that ideally include each of these parameters, as well as more detailed information on patient perianesthetic and postanesthetic outcomes.

Most of these details which could be remedied by more extensive reporting of the operating room procedures, detailed outcomes, in particular longer‐term outcomes, are frequently lacking. Given that there is a real possibility that anesthetic procedures can impact disease progression, as we've seen in the mouse model of LS [8, 9], studies aimed at following long term recovery and disease course are warranted. As long term recovery and disease progression can extend far beyond the perianesthetic period, this would require close collaboration between anesthesiologist, surgeons, and other physicians managing patient care and monitoring disease progression. The ideal follow‐up study might be a multi‐institutional case series of patients monitored for disease progression with a specific consideration of data collection before and after anesthetic exposures. Such a study should include pre‐ and post‐operative measures of underlying disease status using clinical scoring systems such as the Newcastle or IMPDS score as well as metabolic status [66]; full reporting of essential data elements during anesthetic procedures such as carrier gas concentration and regular measures of physiologic status throughout the perianesthetic period; etc. Such datasets could be collected using disease natural history approaches and could reasonably be included in existing natural history studies and linked to existing registries. While collecting data of this quality for a large number of patients is not a trivial task, the insights provided could have a positive impact on anesthesia/procedure risk management in GMD patients.

## Funding

SCJ was supported by an Academy of Medical Sciences Professorship. BMJ was supported by a Lily Foundation grant.

## Supporting information


**Data S1:** Category 3 patient information. A summary of complications detailed in case reports of anesthetic procedures in GMD patients. TIVA—total intravenous anesthesia. VA –general anesthesia with volatile anesthetics. KSS—Kearns‐Sayre Syndrome. MELAS—mitochondrial encephalopathy, lactic acidosis, and stroke‐like episodes. PDH—pyruvate dehydrogenase. FA—Friedreich's Ataxia. AMA—anti‐mitochondrial antibody. LHON—Leber's hereditary optic neuropathy. LS—Leigh Syndrome. HCM—hypertrophic cardiomyopathy. DC—direct current. CT—computed tomography. MRI—magnetic resonance imaging.

**Table S1:** Complication Rates by Clinical Diagnosis. An overview of clinical diagnoses and complication rates by clinical diagnosis among case reports for anesthesia exposures. Male and female patients as indicated (M, F). LS – Leigh syndrome. KSS – Kearns‐Sayre syndrome. MELAS – mitochondrial encephalopathy, lactic acidosis, and stroke‐like episodes. FA – Friedrich's ataxia. LHON – Leber's hereditary optic neuropathy. MNGIE – mitochondrial neuro‐gastrointestinal encephalopathy syndrome. PDH – Pyruvate dehydrogenase deficiency. POLG – Polymerase gamma deficiency. MERFF – myoclonic epilepsy with ragged red fibers. NS – not specified.
**Table S2:** Anesthetic Procedures by Clinical Diagnosis. KSS – Kearns‐Sayre Syndrome. MELAS – mitochondrial encephalopathy, lactic acidosis, and stroke‐like episodes. PDH – pyruvate dehydrogenase. FA – Freidrich's Ataxia. MNGIE – mitochondrial neurogastrointestinal encephalopathy syndrome. MERFF – myoclonic epileopsy with ragged red fibers. POLG – gnee that encodes a DNA polymerase subunit enzyme (gamma); disease with mutation in this gene. LHON – Leber's hereditary optic neuropathy. LS – Leigh Syndrome. RTA – road traffic accident. AEP – auditory envoked potential.
**Table S3:** Procedure lengths. Procedures were grouped into categories based on target organ system. See supplemental data file for all details of specific procedures. Procedure length notes the distribution of procedure length (minimum to maximum) for each category. The number of cases, number of cases reporting complications, and complication rate for each category are provided. Only those with at least 5 cases are plotted in Figures 2 and 3.
**Table S4:** Pharmacologic agents used in TIVA cases. A summary of pharmacologic agents used in TIVA studies for induction and/or maintenance. Total case number and fraction of cases where each drug was reported are provided, as well as the number of cases with reported complications. References for the relevant case report(s) are provided.
**Table S5:** Pharmacologic agents used in VA cases. A summary of pharmacologic agents used in VA studies for induction and/or maintenance. Total case number and fraction of cases where each drug was reported are provided, as well as the number of cases with reported complications. References for the relevant case report(s) are provided.

## Data Availability

The data that supports the findings of this study are available in the Supporting Information of this article.
