# Supplementary material for: Perianesthetic Complications in Genetic Mitochondrial Disease: A Review of Case Reports
Source: Paediatr Anaesth. 2026 Jul 7;36(10):1217–30. doi: 10.1002/pan.70257 (PMC13551163; doi:10.1002/pan.70257)
Supplement: Supplementary file 1 [file PAN-36-1217-s001.docx]

| **Anesthesia Type** | **Diagnosis** | **Procedure Type** | **History** | **Complications** | **Duration (min)** | **Year** | **Citation** |
| --- | --- | --- | --- | --- | --- | --- | --- |
| TIVA | Leigh | Musculoskeletal – muscle biopsy | 14 year old Male  This patient had a history of delayed motor function, respiratory issues, generalized hypotonia, and decreased speech beginning around 6 months of age. This patient was diagnosed after this procedure. | Complications included pyrexia, inspiratory stridor, hyperlactatemia, hyperpyruvatemia, and obtundation. The patient required reintubation 12 hours post procedure. The patient gradually improved over several weeks in hospital but eventually had tracheostomy and gastrostomy procedures for supportive care. The patient remained hospitalized for 1 year before death due to hypoventilation | Not reported | 1990 | [10] |
| TIVA | Mito encephalopathy | Respiratory – lung wedge resection due to neoplasia | 50 year old  Female  This patient was diagnosed prior to this procedure. The patient had a history of progressivexternal ophthalmoplegia. The patient had a lung cancer diagnosis with a mediastinal tumor that had been previously resected | Complications included profound hypotension, sinus bradycardia, ventricular tachycardia, and subsequent cardiac arrest. The patient was administered a DC cardioversion and was diagnosed with stress/non-ischemic cardiomyopathy of unknown cause. The cause of the cardiac arrest is unknown. The patient recovered uneventfully | Not reported | 2013 | [11] |
| TIVA | Unspecified | Oral – endodontic treatment of a necrotic tooth | 10 year old Male  This patient was diagnosed prior to this procedure. The patient had a history of  dysautonomia related to temperature regulation issues, postural orthostatic tachycardia syndrome, syncope, generalized hypotonia, failure to thrive, arthralgia, myalgia, hypermobility of joints, and chronic mild metabolic acidosis. The patient had three prior general anesthetic procedures that were uneventful | Complications included hyperglycemia, transient supraventricular tachycardia, a brief sinus block with return to normal sinus rhythm, and two episodes of apnea. The cardiac abnormalities a total of three times throughout the procedure, self correcting without intervention each time with at total duration of 2 minutes | 160 | 2017 | [12] |
| TIVA | Unspecified | Abdominal – combined pancreatic and renal transplant | 14 year old Female  This patient was diagnosed before this procedure. The patient had a history of  diabetes mellitus, adrenal insufficiency, right sided heart strain with normal output, terminal renal failure, hypothyroidism, hypotonia, ptosis, facial myopathy, pigmentary retinopathy, glaucoma, HCM, encephalopathy, ataxia, a severe ventilatory disorder, increased lacatate, mixed acidosis, hypoglycemia, mild thrombocytopenia, and elevated K and creatinine | Complications included hypotension, tachycardia, severe respiratory depression, nosocomial pneumonia that developed 5 days post-op, septicemia due to duodenojejunostomy anastomosis failure 3-4 weeks post-op. Death occurred 6 weeks post-op due to uncontrollable sepsis | 480 | 2004 | [13] |
| TIVA | Unspecified | Musculoskeletal – muscle biopsy | 4 week old Female  The patient had a history of hypotonia,  somnolence, poor feeding, poor weight gain, a metabolic acidosis, and hyperlactatemia. An MRI performed revealed hyperintensity in the bilateral thalami, basal ganglia, and frontal lobe white matter consistent with a mitochondrial myopathy.  This patient was diagnosed after this procedure | Complications included a metabolic acidosis, hyperlactatemia, and a sinus bradycardia. 3 days post-op the patient became more somnolent and developed a persistent sinus bradycardia which was presumed to be due to ongoing worsening metabolic acidosis. This patient died 2 weeks post-op after being removed from mechanical ventilation | Not reported | 2013 | [14] |
| VA | KSS | Abdominal – exploratory laparotomy due to suspected appendicitis | 51 year old Male  This patient was diagnosed before the procedure. The patient had a history of cardiac  palpitations and dyspnea on mild exertion, peripheral edema, slight proximal muscle weakness, bilateral sensorineural hearing loss; elevated white blood cell counts, slight hepatic dysfunction, and hepatomegaly. The patient had a pacemaker for complete atrioventricular block and atrial tachycardia. Chest radiographs showed enlargement of the cardiothoracic ratio and echocardiography revealed biventricular enlargement and diffuse hypokinesia as well as a decrease in left ventricular ejection fraction, diagnostic for dilated cardiomyopathy | Upon arrival to the ICU for abdominal pain the patient complained of dyspnea and became cyanotic and unresponsive. After intubation, an ECG showed ventricular tachycardia consistent with a rate-limited left bundle-branch block. ICU management stabilised the patient and they were  extubated the next day. The cause of the respiratory depression is unknown | 55 | 1995 | [29] |
| VA | PDH Deficiency | Abdominal - liver biopsy, gastrostomy, and Nissen fundoplication | 10 month old Female  This patient was diagnosed prior to the procedure. The patient had a history of lactic acidosis  Despite intervention base deficit and lactate  progressive deterioration with septicaemia, respiratory distress, and seizures began four days after tracheostomy and the patient died on the 30th day of hospitalisation | On admission, the patient became comatose, developed an irregular respiratory pattern and distress. She was admitted with agonal respiration and unresponsive.  Despite intervention,  base deficit levels increased, there was hyperlactatemia, and progressive deterioration with septicemia occurred. 4 days post op the patient experienced seizures and respiratory distress. The patient died 30 days after being admitted | Not reported | 1983 | [30] |
| VA | MELAS | Endocrine - Thyroid lobectomy | 44 year old Female  This patient was diagnosed prior to the procedure. The patient had a history of  strokes, lactic acidosis, hearing loss, short stature, and IgA nephropathy | Complications included perioperative development of upper airway narrowing with bilateral recurrent laryngeal nerve paralysis and the patient required mechanical ventilation. Post-operatively the patient developed a surgical site hematoma and an infection and required a tracheostomy 15 days post-op | Not reported | 2016 | [16] |
| VA | Mito Depletion | Abdominal – liver transplant | 5 year old Male  This patient was diagnosed prior to the procedure. The patient had a history of general weakness, abnormal movement of the lower extremities, tricuspid regurgitation, liver cirrhosis, liver deficiency, familial intrahepatic cholestasis, and had a cholecystocolonic anastomosis | During the procedure the patient developed hypotension, metabolic acidosis, hyperlactatemia, and hyperglycemia. The patient experienced post-reperfusion syndrome but recovered and was discharged 28 days post-op | 605 | 2024 | [31] |
| VA | AMA positive myositis | Musculoskeletal - Bipolar hip arthroplasty | 66 year old Female  This patient was diagnosed prior to this procedure. The patient had a history of  proximal muscle weakeness, osteonecrosis of the femoral head (steriod presumed), paroxysmal atrial fibrillation, supraventricular arrhythmia, left ventricular dysfunction, chronic heart failure, and sinus arrest had occurred twice after supraventricular tachycardia | Complications included a severe bradycardia, an AV block, supraventricular tachycardia after the start of anesthesia, and sinus arrest 60 minutes into the procedure that lasted 9000ms and spontaneously resolved | Not reported | 2023 | [32] |
| VA | FA | Musculoskeletal - Spinal fusion, scoliosis correction | 20 year old Female  This patient was diagnosed prior to the procedure. The patient had a history of a systolic ejection murmur, muscle weakness, incoordination, and progressive thoracolumbar kyphoscoliosis | Complications included supraventricular tachycardia which resolved spontaneously, hypertension, hypothermia, and a marked metabolic acidosis | 240 | 1984 | [33] |
| VA | MELAS | Cardiac – cardiac transplant | 30 year old Female  This patient was diagnosed prior to the procedure. The patient had a history of malnutrition, muscle wasting, strokes, left hemiparesis, gait imbalance, myopathy, seizures, short term memory loss, migraines, hypertrophy cardiomyopathy with biatrial enlargement,  pericardial effusion, congestive heart failure, and pulmonary hypertension | Complications included post-op adrenal insufficiency and acute renal failure | 540 | 2011 | [24] |
| VA | MELAS | Abdominal – colectomy, celiotomy | 65 year old Male  This patient was diagnosed prior to the procedure. The patient had a history of  Cardiac regional wall motion abnormalities, pleural effusion, left bundle branch block, diabetes mellitus, autonomic neuropathy, megacolon, intestinal pseudo-obstruction, malnutrition, chronic diarrhea, and hearing loss | Complications included intraoperative ventricular tachycardia, hypotension, intra-abdominal septicemia, and hyperglycemia | 247 | 2011 | [24] |
| VA | Leigh | Musculoskeletal - Spinal cord malformation correction - palliative | 21 year old Female  This patient was diagnosed prior to the procedure. The patient had a history of  non-verbal communication, poor swallowing reflexes, and a weak cough | Complications included clinical deterioration with pulmonary consolidation requiring the patient to be re-intubated 1 hour post-op, suspected septicemia, apnea, cerebral and ventricular changes with new progressive necrosis noted on serial MRI. The patient required cardiovascular support and a. tracheostomy 16 days post op as they developed frequent apneic periods with spontaneous respiratory effort. The patient died 62 days post op | 420 | 2003 | [34] |
| VA | Leigh | Musculoskeletal – muscle biopsy | 3 year old Female  The patient had a history of  hypotonia, ataxia, developmental delay, nystagmus, hyperlactataemia, and other CNS symptoms. This patient was diagnosed after the procedure | Complications included a marked mixed metabolic and respiratory acidosis, lactic acidosis, hyperglycemia, hyperventilation, and tachycardia throughout the procedure. The patient had an episode of what looked like malignant hyperthermia | Not reported | 1996 | [21] |
| VA | Leigh | Brain - pneumoencephalogram | 21 year old Male  The patient had a history of hypotonia, lethargy, an abnormal gait, mostly non-verbal, strabismus, nystagmus, and bilateral optic atrophy | Complications included apnea, hypertension, and seizures. The patient died 34 hours post-op and a post-mortem revealed pulmonary edema, tracheitis, bronchiolitis, and typical LS brain lesions | Not reported | 1990 | [10] |
| VA | Leigh | Respiratory - bronchoscopy | 4 year old Female  The patient had a history of  severe delay in motor development and respiratory issues. This patient was diagnosed after the procedure | Complications included apnea, hypotonia, stridor, hyperlactatemia, and hyperpyruvatemia. The patient required reintubation 48 hours post-op. The patient was noted to have ptosis and bilateral optic atrophy and a CT scan performed revealed brain lesions. The patient died 5 days post-op | Not reported | 1990 | [10] |
| VA | Leigh/LHON | Otic – surgical correction of bilateral tympanostomy tube placement | 18 month old Male  The patient had a history of normal development until 12 months of age and rapid eye movement. This patient was diagnosed after this procedure | Complications included significant clinal deterioration post-op, ataxia, opsoclonus, myoclonus, and language and motor regression. Further deterioration occurred following a later anesthesia but three subsequent additional exposures with more care given to perioperative maintenance showed no further regression | Not reported | 2019 | [35] |
| VA | Unspecified mito encephalopathy | Abdominal – renal transplant | 52 year old Male  This patient was diagnosed before this procedure. The patient had a history of  muscle weakness, deafness, cerebellar ataxia, diabetes mellitus, and renal failure | Complications included respiratory depression and hypotension. The patient recovered uneventfully | Not reported | 2009 | [36] |
| VA | Unspecified mito encephalopathy | Dental – maxillary cyst removal | 21 year old Male  This patient was diagnosed prior to the procedure. No patient history was provided in the case report | Complications included apnea after fentanyl administrationand respiratory depression | Not reported | 2006 | [37] |

**Category 3 patient information.** A summary of complications detailed in case reports of anesthetic procedures in GMD patients. TIVA – total intravenous anesthesia. VA –general anesthesia with volatile anesthetics. KSS – Kearns-Sayre Syndrome. MELAS – mitochondrial encephalopathy, lactic acidosis, and stroke-like episodes. PDH – pyruvate dehydrogenase. FA – Friedreich’s Ataxia. AMA – anti-mitochondrial antibody. LHON – Leber’s hereditary optic neuropathy. LS – Leigh Syndrome. HCM – hypertrophic cardiomyopathy. DC – direct current. CT – computed tomography. MRI – magnetic resonance imaging.
