# Supplementary material for: Perianesthetic Complications in Genetic Mitochondrial Disease: A Review of Case Reports
Source: Paediatr Anaesth. 2026 Jul 7;36(10):1217–30. doi: 10.1002/pan.70257 (PMC13551163; doi:10.1002/pan.70257)
Supplement: Supplementary file 2 [file PAN-36-1217-s002.docx]

**Tables**

***Supplemental Table 1***

| **Clinical diagnosis** | **All Patients** | **Patients with Complications** | **Complication**  **rate (%)** |
| --- | --- | --- | --- |
| Unspecified mitochondrial disease | 10 (4M, 6F) | 5 (2M, 3F) | 50.0% |
| LS | 23 (10M, 13F) | 9 (3M, 6F) | 39.1% |
| OTHER | 20 (10M, 9F, 1NS) | 7 (4M, 3F) | 35.0% |
| KSS | 13 (8M, 5F) | 4 (3M, 1 F) | 30.8% |
| MELAS | 33 (14M, 18F, 1NS) | 8 (5M, 3F) | 24.2% |
| Mitochondrial encephalomyopathy | 24 (11M, 13F) | 4 (2M, 2F) | 16.7% |
| FA | 12 (0M, 11F, 1NS) | 1 (0M, 1F) | 8.3% |
| *Fewer than 10 cases* |  |  |  |
| LHON | 1 (M) | 1 (1M, 0F) | 100% |
| MNGIE | 2 (1M, 1F) | 1 (1M, 0F) | 50% |
| PDH | 4 (3M, 1F) | 2 (1M, 1F) | 50% |
| POLG | 3 (1M, 2F) | 1 (1M, 0F) | 33.3% |
| MERFF | 3 (2M, 1F) | 1 (1M, 0F) | 33.3% |

**Supplemental Table 1 - Complication Rates by Clinical Diagnosis.** An overview of clinical diagnoses and complication rates by clinical diagnosis among case reports for anesthesia exposures. Male and female patients as indicated (M, F). LS – Leigh syndrome. KSS – Kearns-Sayre syndrome. MELAS – mitochondrial encephalopathy, lactic acidosis, and stroke-like episodes. FA – Friedrich’s ataxia. LHON – Leber’s hereditary optic neuropathy. MNGIE – mitochondrial neuro-gastrointestinal encephalopathy syndrome. PDH – Pyruvate dehydrogenase deficiency. POLG – Polymerase gamma deficiency. MERFF – myoclonic epilepsy with ragged red fibres. NS – not specified.

***Supplemental Table 2***

| **Disease Type:** | Cardiac | Abdominal | Respiratory | Genitourinary | Musculoskeletal | Otic | Ophthalmic | Dental | Brain | Dermal | Endocrine | Other |
| --- | --- | --- | --- | --- | --- | --- | --- | --- | --- | --- | --- | --- |
| KSS (13) | 5 | 2 | 1 | 1 |  |  | 1 |  | 1 | 2 |  |  |
| PDH (6) |  | 2 | 2 | 1 | 1 |  |  |  |  |  |  |  |
| MELAS (34) | 3 | 14 | 2 |  | 2 | 4 | 1 | 2 | 3 |  | 3 |  |
| Mitochondrial encephalomyopathy (24) | 1 | 11 | 4 | 1 |  | 1 | 4 | 1 |  |  | 1 |  |
| LS (19) |  | 3 | 2 | 1 | 6 | 1 | 2 | 3 | 1 |  |  |  |
| POLG (3) |  | 1 |  |  |  |  |  |  | 2 |  |  |  |
| MERFF (3) |  |  |  |  | 1 |  |  |  |  | 2 |  |  |
| FA (12) | 1 | 1 |  | 2 | 8 |  |  |  |  |  |  |  |
| LHON (1) |  |  |  |  |  |  |  |  |  |  |  | 1 (RTA) |
| MNGIE (3) |  | 2 |  | 1 |  |  |  |  |  |  |  |  |
| Unspecified mito (10) | 2 | 3 |  |  | 1 |  |  | 3 |  |  |  | 1 (AEP test) |
| Other (21) | 4 | 7 |  |  | 5 | 3 |  | 1 |  |  |  |  |

**Supplemental Table 2 - Anesthetic Procedures by Clinical Diagnosis.** KSS – Kearns-Sayre Syndrome**.** MELAS – mitochondrial encephalopathy, lactic acidosis, and stroke-like episodes**.** PDH – pyruvate dehydrogenase**.** FA – Freidrich’s Ataxia**.** MNGIE – mitochondrial neurogastrointestinal encephalopathy syndrome**.** MERFF – myoclonic epileopsy with ragged red fibres**.** POLG – gnee that encodes a DNA polymerase subunit enzyme (gamma); disease with mutation in this gene**.** LHON – Leber’s hereditary optic neuropathy**.** LS – Leigh Syndrome**.** RTA - road traffic accident**.** AEP – auditory envoked potential

***Supplemental Table 3***

| **Procedures** | **Procedure length** | **Cases** | **Cases with complications** | **Complication**  **Rate** |
| --- | --- | --- | --- | --- |
| Musculoskeletal | 45-420 min | 24 | 11 | 31.43% |
| Respiratory | 20-326 min | 9 | 3 | 25% |
| Brain | 43-220 min | 5 | 2 | 40% |
| Abdominal | 9-605 min | 48 | 18 | 27.27% |
| Cardiac | 44-540 min | 17 | 4 | 19.05% |
| Dental | 16-300 min | 13 | 2 | 13.33% |
| Ophthalmic | 47-189 min | 7 | 1 | 12.50% |
| Otic | 40-306 min | 12 | 2 | 14.29% |
| Dermal | 170-300 min | 5 | 1 | 16.67% |
| ***Less than 5 cases*** |  |  |  |  |
| *Genitourinary* | *15-110 min* | *4* | *0* | 0% |
| *Endocrine* | *Not reported* | *2* | *1* | 33.33% |
| *Polytrauma* | *Not reported* | *2* | *0* | 0% |

**Supplemental Table 3 - Procedure lengths.** Procedures were grouped into categories based on target organ system. See supplemental data file for all details of specific procedures. Procedure length notes the distribution of procedure length (minimum to maximum) for each category. The number of cases, number of cases reporting complications, and complication rate for each category are provided. Only those with at least 5 cases are plotted in ***Figure 2*** and ***Figure 3***.

***Supplemental Table 4***

| **Pharmacologic agents used in TIVA (78 cases)** | | | | | | |
| --- | --- | --- | --- | --- | --- | --- |
|  | **Cases** | **Percent of all TIVA cases** | **Induction** | **Maintenance** | **Cases with Complications** | **Citations** |
| Propofol | 58 | 74.4% | 54 | 52 | 12 | [1-25] |
| Etomidate | 2 | 2.6% | 2 | 0 | 1 | [26] |
| N_2_O | 20 | 25.6% | 4 | 17 | 1 | [11, 26-40] |
| Ketamine | 13 | 16.7% | 11 | 7 | 1 | [6, 12, 13, 18, 26, 28, 37, 41-46] |
| Dexmedetomidine | 7 | 9.0% | 4 | 7 | 2 | [26, 39, 41, 43, 46-48] |
|  |  |  |  |  |  |  |
| *Opiods* |  |  |  |  |  |  |
| Fentanyl | 35 | 44.9% | 34 | 15 | 5 | [2, 5, 6, 10, 11, 14, 15, 17, 19-21, 23, 26-29, 38, 39, 41, 42, 44, 46, 49-51] |
| Remifentanil | 24 | 30.8% | 9 | 23 | 6 | [1, 2, 4, 7-10, 14, 16, 17, 24-26, 28, 30, 39, 45, 47, 51-56] |
| Alfentanil | 6 | 7.7% | 5 | 6 | 1 | [22, 33, 34, 57-59] |
| Sufentanil | 5 | 6.4% | 4 | 3 | 0 | [31, 46, 55, 60] |
| Hydromorphone | 1 | 1.3% | 1 | 0 | 0 | [1] |
| Piritramide | 1 | 1.3% | 1 | 0 | 0 | [33] |
| Droperidol | 2 | 2.7% | 1 | 1 | 0 | [27, 61] |
|  |  |  |  |  |  |  |
| *Benzodiazipines* |  |  |  |  |  |  |
| Diazepam | 5 | 6.4% | 5 | 0 | 2 | [1, 29, 33, 34, 62, 63] |
| Midazolam | 21 | 26.9% | 20 | 3 | 2 | [1, 5, 6, 14, 15, 17, 18, 22, 26, 27, 30, 37, 39, 40, 47, 50, 53, 63-65] |
| Remimazolam | 3 | 3.9% | 3 | 3 | 1 | [51, 54, 56] |
| Lorazepam | 1 | 1.3% | 1 | 0 | 0 | [18, 29] |
| Chlorazepam | 1 | 1.3% | 1 | 0 | 1 | [29] |
| Temazepam | 1 | 1.3% | 1 | 0 | 0 | [32] |
| Clorazepate | 2 | 2.6% | 2 | 0 | 0 | [60] |
|  |  |  |  |  |  |  |
| *Barbituates* |  |  |  |  |  |  |
| Thiopental | 3 | 3.9% | 1 | 2 | 0 | [18, 27, 61] |
| Methohexital | 1 | 1.3% | 1 | 1 | 0 | [46] |
|  |  |  |  |  |  |  |
| *NMBs* |  |  |  |  |  |  |
| Vecuronium | 10 | 12.8% | 10 | 3 | 2 | [6, 20, 21, 33, 42, 59, 61, 64, 66] |
| Rocuronium | 20 | 25.6% | 18 | 6 | 5 | [2, 3, 5, 7, 9-11, 14, 17, 19, 25-29, 43, 44, 51, 53] |
| Cisatracurium | 6 | 7.7% | 6 | 3 | 1 | [4, 23, 46, 52, 57, 65] |
| Mivacurium | 1 | 1.2% | 1 | 1 | 0 | [15] |
| Atracurium | 3 | 3.9% |  |  | 0 | [16, 32, 40] |
|  |  |  |  |  |  |  |
| *Anticholinergics* |  |  |  |  |  |  |
| Atropine | 5 | 6.4% | 5 | 0 | 0 | [2, 34, 36, 37, 50] |
| Glycopyrrolate | 3 | 3.9% | 3 | 0 | 1 | [12, 60] |
|  |  |  |  |  |  |  |
| *Local anesthetics* |  |  |  |  |  |  |
| Ropivacaine | 1 | 1.3% | 0 | 1 | 1 | [21] |
| Lidocaine | 8 | 10.3% | 7 | 1 | 1 | [1, 16, 22, 27, 31, 47, 52, 55] |
| Bupivacaine | 2 | 2.6% | 2 | 1 | 1 | [48, 51] |
|  |  |  |  |  |  |  |
| *GI Drugs* |  |  |  |  |  |  |
| Metoclopramide | 1 | 1.3% | 1 | 0 | 0 | [36] |
| Ranitidine | 1 | 1.3% | 1 | 0 | 0 | [36] |
|  |  |  |  |  |  |  |
| *Misc.* |  |  |  |  |  |  |
| Phenytoin | 2 | 2.6% | 2 | 0 | 0 | [13, 18] |
| Diphenhydramine | 1 | 1.3% | 1 | 0 | 1 | [47] |
| Verapamil | 1 | 1.3% | 1 | 0 | 0 | [32] |

**Supplemental Table 4 *–* Pharmacologic agents used in TIVA cases.** A summary of pharmacologic agents used in TIVA studies for induction and/or maintenance. Total case number and fraction of cases where each drug was reported are provided, as well as the number of cases with reported complications. References for the relevant case report(s) are provided. See supplemental data file for additional details.

***Supplemental Table 5***

| **Pharmacologic agents used in VA (77 cases)** | | | | | | | |
| --- | --- | --- | --- | --- | --- | --- | --- |
|  | **Cases** | **Percent of all VA cases** | **Induction** | **Maintenance** | **Cases with Complications** | **Citations** |  |
| N_2_O | 41 | 53.3% | 19 | 26 | 19 | [67-91] |  |
| Propofol | 41 | 53.3% | 37 | 10 | 19 | [73, 74, 76, 78, 79, 81, 84, 87, 88, 90-104] |  |
| Etomidate | 5 | 6.5% | 1 | 0 | 2 | [68, 73, 92, 105, 106] |  |
| Ketamine | 6 | 7.8% | 6 | 2 | 4 | [73, 103, 106-109] |  |
| Dexmedetomidine | 3 | 3.9% | 2 | 2 | 1 | [83, 107, 109] |  |
|  |  |  |  |  |  |  |  |
| *Volatile Anaesthetics* |  |  |  |  |  |  |  |
| Sevoflurane | 35 | 45.5% | 21 | 28 | 12 | [68, 73-75, 81, 83, 85, 87, 88, 91, 92, 95, 96, 98, 100-104, 106-108, 110-112] |  |
| Isoflurane | 17 | 22.1% | 5 | 13 | 6 | [69, 70, 73, 80, 82, 84, 86, 89, 113-115] |  |
| Halothane | 10 | 13% | 4 | 6 | 6 | [72, 73, 79, 105, 116-118] |  |
| Desflurane | 12 | 15.6% | 7 | 6 | 6 | [71, 73, 93, 94, 97, 109, 119] |  |
| Enflurane | 2 | 2.6% | 0 | 2 | 0 | [120] |  |
|  |  |  |  |  |  |  |  |
| *Opiods* |  |  |  |  |  |  |  |
| Fentanyl | 42 | 54.6% | 38 | 10 | 17 | [68, 70, 71, 76, 80, 83, 84, 86, 90, 93, 96-101, 106-110, 112, 115, 119, 120] |  |
| Remifentanil | 14 | 18.2% | 5 | 13 | 5 | [74, 78, 81, 83, 85, 87, 92, 93, 101, 102, 104, 109, 111] |  |
| Sufentanil | 4 | 5.2% | 4 | 2 | 2 | [92, 103, 116, 121] |  |
| Alfentanil | 3 | 3.9% | 0 | 3 | 2 | [79, 107] |  |
| Papaveretum | 3 | 3.9% | 3 | 0 | 3 | [72] |  |
| Droperidol | 3 | 3.9% | 3 | 0 | 1 | [113, 120] |  |
| Levorphanol | 2 | 2.6% | 2 | 0 | 0 | [105, 117] |  |
| Morphine | 2 | 2.6% | 2 | 0 | 1 | [113, 115] |  |
| Thalamonal | 1 | 1.3% | 1 | 0 | 0 | [89] |  |
| Meperidine | 1 | 1.3% | 0 | 1 | 0 | [118] |  |
|  |  |  |  |  |  |  |  |
| *Benzodiazipines* |  |  |  |  |  |  |  |
| Midazolam | 32 | 41.6% | 32 | 4 | 14 | [70, 71, 75, 79, 82, 84, 85, 90-92, 96, 100, 106-109, 115, 119] |  |
| Diazepam | 6 | 7.8% | 6 | 0 | 1 | [86, 89, 101, 116, 120] |  |
|  |  |  |  |  |  |  |  |
| *Barbituates* |  |  |  |  |  |  |  |
| Thiopental/thiopentone | 16 | 20.8% | 13 | 3 | 7 | [69, 70, 72, 73, 77, 80, 89, 113, 114, 116-118, 120, 121] |  |
| Thiamylal | 1 | 1.3% | 1 | 0 | 0 | [112] |  |
|  |  |  |  |  |  |  |  |
| *NMBs* |  |  |  |  |  |  |  |
| Rocuronium | 16 | 20.8% | 14 | 3 | 7 | [77, 85, 91, 92, 99-103, 106, 109, 111, 119, 121] |  |
| Vecuronium | 13 | 16.9% | 11 | 2 | 6 | [79, 80, 94, 116, 122] |  |
| Atracurium | *8* | 10.4% | 8 | 1 | 0 | [67, 84, 86, 87, 98, 120] |  |
| Cisatricurium | *7* | 9.1% | 6 | 2 | 3 | [81, 87, 92, 96, 112] |  |
| Pancuronium | *5* | 6.5% | 5 | 0 | 4 | [70, 113, 114] |  |
| Suxamethionine /succinylcholine | *5* | 6.5% | 4 | 1 | 3 | [72, 105] |  |
| Tubocurarine | *2* | 2.6% | 2 | 1 | 1 | [118, 120] |  |
| Curare | *1* | 1.3% | 1 | 0 | 0 | [86] |  |
|  |  |  |  |  |  |  |  |
| *Anticholinergics* |  |  |  |  |  |  |  |
| Atropine | 8 | 10.4% | 7 | 1 | 3 | [82, 89, 91, 101, 105, 112, 117, 118] |  |
| Scopolamine/Hyoscine | 7 | 9.1% | 7 | 0 | 5 | [72, 80, 105, 113, 117] |  |
|  |  |  |  |  |  |  |  |
| *Local anaesthetics* |  |  |  |  |  |  |  |
| Lidocaine | 4 | 5.2% | 4 | 0 | 2 | [71, 93, 96, 104] |  |
| Bupivacaine | 2 | 2.6% | 1 | 1 | 0 | [67, 110] |  |
| Ropivacaine | 1 | 1.3% | 1 | 0 | 1 | [97] |  |
|  |  |  |  |  |  |  |  |
| *Misc.* |  |  |  |  |  |  |  |
| Hydroxyzine | 1 | 1.3% | 1 | 0 | 1 | [80] |  |
| Promethazine | 1 | 1.3% | 1 | 0 | 0 | [115] |  |
| Acetaminophen | 1 | 1.3% | 1 | 0 | 0 | [108] |  |
| Carnitine | 1 | 1.3% | 1 | 0 | 1 | [101] |  |
| Clonidine | 1 | 1.3% | 1 | 0 | 0 | [67] |  |
| Ranitidine | 2 | 2.6% | 2 | 0 | 0 | [84] |  |

**Supplemental Table 5 - Pharmacologic agents used in VA cases.** A summary of pharmacologic agents used in VA studies for induction and/or maintenance. Total case number and fraction of cases where each drug was reported are provided, as well as the number of cases with reported complications. References for the relevant case report(s) are provided. See supplemental data file for additional details.

1.	Agamez Medina, G.L., et al., *Anaesthesia for correction of scoliosis in pediatric patient with Friedreich's ataxia.* Rev Esp Anestesiol Reanim, 2015. **62**(1): p. 42-5.

2.	Fedriani, J.J., *[Anesthetic management of a patient with mitochondrial encephalomyopathy underwent emergency open cholecystectomy].* Rev Esp Anestesiol Reanim, 2014. **61**(8): p. 471-2.

3.	Fujita, T., et al., *[Anesthetic Management of Laparoscopic Cholecystectomy in a Patient with Mitochondrial Encephalomyopathy].* Masui, 2017. **66**(2): p. 164-167.

4.	Gentili, M.E., et al., *Combination of general anaesthesia and postoperative epidural analgesia in mitochondrial myopathy.* Ann Fr Anesth Reanim, 2013. **32**(10): p. e149.

5.	Ibanez, C. and I. Fernandez-Gonzalez, *[Emergency anesthesia in a woman with mitochondrial neurogastrointestinal encephalopathy].* Rev Esp Anestesiol Reanim, 2011. **58**(9): p. 585-7.

6.	Imai, Y., et al., *[Anesthetic management of emergency total gastrectomy in a patient with mitochondrial encephalomyopathy: a case of gastric perforation accompanied by systemic inflammatory response syndrome].* Masui, 2010. **59**(6): p. 765-9.

7.	Kawagoe, I., et al., *[A case of intraoperative cardiac arrest in a patient with mitochondrial encephalomyopathy undergoing lung resection].* Masui, 2013. **62**(4): p. 431-4.

8.	Kilic, E.T., et al., *Anesthetic Management in Pediatric Patient for Percutaneous Endoscopic Gastrostomy with Mitochondrial Myopathy: Leigh Syndrome.* Anesth Essays Res, 2018. **12**(1): p. 276-278.

9.	Kim, J.Y., et al., *Anesthetic considerations of Joubert syndrome in patients with mitochondrial disease - A case report.* Anesth Pain Med (Seoul), 2021. **16**(2): p. 158-162.

10.	Kism, M., et al., *[Anesthetic Management for Pacemaker Implantation in a Child with Mitochondrial Diseases and Complete Atrioventricular Block].* Masui, 2016. **65**(9): p. 955-960.

11.	Lilitsis, E., et al., *Anesthetic management of a pediatric patient with Electron Transfer Flavoprotein Dehydrogenase deficiency (ETFDH) and acute appendicitis: case report and review of the literature.* BMC Anesthesiol, 2017. **17**(1): p. 116.

12.	Maddali, M.M., et al., *Propofol and Kearns-Sayre Syndrome: An idiographic approach.* Sultan Qaboos Univ Med J, 2023. **23**(Spec Iss): p. 63-67.

13.	Marquardt, L., et al., *No effect of electrical transcranial direct current stimulation adjunct treatment for epilepsia partialis continua in POLG disease.* Epilepsy Behav Rep, 2019. **12**: p. 100339.

14.	Motegi, M., et al., *Cochlear Implantation for Isoleucyl-tRNA Synthetase Mutation-Associated Mitochondrial Disease: A Case Report.* Cureus, 2024. **16**(8): p. e67760.

15.	Ortiz-Gomez, J.R. and J.M. Souto-Ferro, *[Anesthesia for a patient with mitochondrial respiratory chain complex III deficiency].* Rev Esp Anestesiol Reanim, 2006. **53**(9): p. 575-9.

16.	Park, J.S., et al., *Total intravenous anesthesia with propofol and remifentanil in a patient with MELAS syndrome -A case report.* Korean J Anesthesiol, 2010. **58**(4): p. 409-12.

17.	Pedroviejo, V., et al., *[Anesthesia during laparoscopic cholecystectomy in a patient with mitochondrial myopathy, encephalopathy, lactic acidosis, and stroke-like episodes (MELAS-syndrome)].* Rev Esp Anestesiol Reanim, 2010. **57**(2): p. 120-2.

18.	Pruss, H. and M. Holtkamp, *Ketamine successfully terminates malignant status epilepticus.* Epilepsy Res, 2008. **82**(2-3): p. 219-22.

19.	Saettele, A.K., A. Sharma, and D.J. Murray, *Case scenario: Hypotonia in infancy: anesthetic dilemma.* Anesthesiology, 2013. **119**(2): p. 443-6.

20.	Sasaki, R., K. Nanjo, and K. Hirota, *Perioperative anesthetic managements for the laryngo-tracheal separation and open fundoplication in a 17-year-old patient with Leigh syndrome.* Paediatr Anaesth, 2008. **18**(11): p. 1133-4.

21.	Sasano, N., et al., *Severe hyponatremia occurring after surgical stress in a patient with mitochondrial disease.* J Anesth, 2009. **23**(4): p. 587-90.

22.	Schnabel, R.M., et al., *Anesthetic management for a child with mitochondrial complex II deficiency.* Paediatr Anaesth, 2008. **18**(8): p. 802-3.

23.	Terkawi, A.S., et al., *Anesthetic considerations in Leigh disease: Case report and literature review.* Saudi J Anaesth, 2012. **6**(2): p. 181-5.

24.	Vanlander, A.V., et al., *Inborn oxidative phosphorylation defect as risk factor for propofol infusion syndrome.* Acta Anaesthesiol Scand, 2012. **56**(4): p. 520-5.

25.	Yamashita, A. and T. Iimori, *[Total intravenous anesthesia for a patient with mitochondrial encephalomyopathy who underwent laparoscopic cholecystotomy].* Masui, 2011. **60**(8): p. 961-3.

26.	Nelson, J.H. and R.F. Kaplan, *Anesthetic Management of Two Pediatric Patients With Concurrent Diagnoses of Mitochondrial Disease and Malignant Hyperthermia Susceptibility: A Case Report.* A A Case Rep, 2017. **9**(7): p. 204-206.

27.	Ciccotelli, K.K., E.L. Prak, and S. Muravchick, *An adult with inherited mitochondrial encephalomyopathy: report of a case.* Anesthesiology, 1997. **87**(5): p. 1240-2.

28.	Farag, E., et al., *The anesthetic management of ventricular septal defect (VSD) repair in a child with mitochondrial cytopathy.* Can J Anaesth, 2002. **49**(9): p. 958-62.

29.	Finsterer, J., et al., *Increased sensitivity to rocuronium and atracurium in mitochondrial myopathy.* Can J Anaesth, 1998. **45**(8): p. 781-4.

30.	Guasch, E., et al., *Progressive external ophthalmoplegia and ambulatory remifentanil-propofol based anaesthesia.* Anaesthesia, 2003. **58**(6): p. 607-8.

31.	Hara, K., T. Sata, and A. Shigematsu, *Anesthetic management for cardioverter-defibrillator implantation in a patient with Kearns-Sayre syndrome.* J Clin Anesth, 2004. **16**(7): p. 539-41.

32.	Kelly, A. and M. O'Connor, *Mitochondrial myopathy and anaesthesia.* Anaesthesia, 1990. **45**(7): p. 596.

33.	Klockgether-Radke, A., et al., *[General anesthesia in two patients with mitochondrial myopathy].* Anaesthesist, 1993. **42**(2): p. 111-4.

34.	Levent, K., G. Yavuz, and T. Kamil, *Anaesthesia for Friedreich's ataxia. Case report.* Minerva Anestesiol, 2000. **66**(9): p. 657-60.

35.	Naguib, M., et al., *Sensitivity to mivacurium in a patient with mitochondrial myopathy.* Anesthesiology, 1996. **84**(6): p. 1506-9.

36.	Sabate, S., et al., *[Anesthesia in Kearns-Sayre syndrome (mitochondrial myopathy)].* Rev Esp Anestesiol Reanim, 1996. **43**(7): p. 255-7.

37.	Shenkman, Z., et al., *Anaesthetic management of a patient with Leigh's syndrome.* Can J Anaesth, 1997. **44**(10): p. 1091-5.

38.	Shibukawa, K., et al., *[The use of propofol combined with nitrous oxide and fentanyl in anesthetic management of a patient with mitochondrial encephalomyopathy].* Masui, 2002. **51**(8): p. 888-91.

39.	Suleman, M.I., et al., *Non-Trigger Anesthesia Management in a Patient With Leigh's Syndrome Presenting for Dental Rehabilitation.* Anesth Pain Med, 2015. **5**(6): p. e28804.

40.	Torres, M.D., et al., *[Anesthesia in a case of mitochondrial myopathy].* Rev Esp Anestesiol Reanim, 1996. **43**(3): p. 114-5.

41.	Ahmed, N., *Anaesthetic management of an infant with MEGD(H)EL syndrome undergoing cochlear implant.* BMC Anesthesiol, 2024. **24**(1): p. 428.

42.	Kushikata, T., et al., *[Total intravenous anesthesia with propofol, ketamine, and fentanyl (PFK) for a patient with mitochondrial myopathy].* Masui, 2004. **53**(2): p. 178-80.

43.	Salik, I., K. Kubal, and S. Barst, *Anesthetic Implications of a Patient With Kniest Dysplasia and Mitochondrial Disease: A Case Report.* A A Pract, 2019. **12**(8): p. 267-269.

44.	Sharma, A.D., et al., *Anaesthetic considerations for a child with combined Prader-Willi syndrome and mitochondrial myopathy.* Paediatr Anaesth, 2001. **11**(4): p. 488-90.

45.	Tan, A.L. and R. Goy, *Anaesthetic management of a patient with Leigh's syndrome with central hypoventilation and obstructive sleep apnoea.* Singapore Med J, 2013. **54**(12): p. e250-3.

46.	Woodward, E.L. and Z. Xiong, *Use of Methohexital and Dexmedetomidine for Maintenance of Anesthesia in a Patient With Mitochondrial Myopathy: A Case Report.* A A Case Rep, 2017. **8**(2): p. 33-35.

47.	Gordon, T.R. and R.J. Montandon, *Anesthetic Management of a Child With Unspecified Mitochondrial Disease in an Outpatient Dental Setting.* Anesth Prog, 2017. **64**(1): p. 33-38.

48.	Sugur, T., et al., *Regional anesthesia in two consecutive surgeries in a patient with mitochondrial neurogastrointestinal encephalomyopathy: a case report.* Braz J Anesthesiol, 2021. **71**(1): p. 84-86.

49.	Tatsumi, Y., et al., *[Anesthetic management of a patient with mitochondrial encephalomyopathy under total intravenous anesthesia].* Masui, 2006. **55**(10): p. 1228-30.

50.	Thiel, A., M. Ritzka, and G. Saur, *[Anesthesia in mitochondrial encephalomyopathies].* Anasthesiol Intensivmed Notfallmed Schmerzther, 2001. **36**(7): p. 437-9.

51.	Yamadori, Y., et al., *General anesthesia with remimazolam for a pediatric patient with MELAS and recurrent epilepsy: a case report.* JA Clin Rep, 2022. **8**(1): p. 75.

52.	Aouad, M.T., F.J. Gerges, and A.S. Baraka, *Resistance to cisatracurium in a patient with MELAS syndrome.* Paediatr Anaesth, 2005. **15**(12): p. 1124-7.

53.	Bolton, P., et al., *Anaesthesia for an adolescent with mitochondrial encephalomyopathy-lactic acidosis-stroke-like episodes syndrome.* Paediatr Anaesth, 2003. **13**(5): p. 453-6.

54.	Kitaura, A., et al., *Remimazolam anesthesia for transcatheter mitral valve repair in a patient with mitochondrial myopathy, encephalopathy, lactic acidosis, and stroke-like episodes (MELAS) syndrome: a case report.* JA Clin Rep, 2022. **8**(1): p. 38.

55.	Pancaro, C. and D. Renz, *Anesthetic management in Friedreich's ataxia.* Paediatr Anaesth, 2005. **15**(5): p. 433-4.

56.	Suzuki, Y., M. Doi, and Y. Nakajima, *General anesthesia with remimazolam in a patient with mitochondrial encephalomyopathy: a case report.* JA Clin Rep, 2021. **7**(1): p. 51.

57.	Heinke, W. and L. Schaffranietz, *[Anaesthesia for combined pancreatic and renal transplantation in a patient with mitochondrial encepahalomyopathy--a case report].* Anaesthesiol Reanim, 2004. **29**(3): p. 87-90.

58.	Singh, B., P.S. Low, and J.F. Yeo, *MELAS: a case report.* Ann Acad Med Singap, 2004. **33**(4 Suppl): p. 69-71.

59.	Wisely, N.A. and P.R. Cook, *General anaesthesia in a man with mitochondrial myopathy undergoing eye surgery.* Eur J Anaesthesiol, 2001. **18**(5): p. 333-5.

60.	Schmitt, H.J., S. Wick, and T. Munster, *Rocuronium for muscle relaxation in two children with Friedreich's ataxia.* Br J Anaesth, 2004. **92**(4): p. 592-6.

61.	Matsuno, S., H. Hashimoto, and A. Matsuki, *[Neuroleptanesthesia for a patient with mitochondrial encephalomyopathy].* Masui, 1994. **43**(7): p. 1038-40.

62.	Cheam, E.W. and L.A. Critchley, *Anesthesia for a child with complex I respiratory chain enzyme deficiency.* J Clin Anesth, 1998. **10**(6): p. 524-7.

63.	Ellis, Z. and C. Bloomer, *Outpatient anesthesia for oral surgery in a juvenile with Leigh disease.* Anesth Prog, 2005. **52**(2): p. 70-3.

64.	Itaya, K., et al., *[Anesthetic management of two patients with mitochondrial encephalopathy, lactic acidosis and stroke-like episodes (MELAS)].* Masui, 1995. **44**(5): p. 710-2.

65.	Thompson, V.A. and J.A. Wahr, *Anesthetic considerations in patients presenting with mitochondrial myopathy, encephalopathy, lactic acidosis, and stroke-like episodes (MELAS) syndrome.* Anesth Analg, 1997. **85**(6): p. 1404-6.

66.	Brustia, L., et al., *[TIVA inmitochondrial myopathy. A clinical case].* Minerva Anestesiol, 1993. **59**(11): p. 611-3.

67.	Acharya, D. and O.R. Dearlove, *Anaesthesia in pyruvate dehydrogenase deficiency.* Anaesthesia, 2001. **56**(8): p. 808-9.

68.	Baldwin, M.K. and V.N. Nembhard, *Anesthetic considerations in a teenager with advanced symptoms of Kearns-Sayre syndrome.* Paediatr Anaesth, 2009. **19**(6): p. 639-40.

69.	Burns, A.M. and M.P. Shelly, *Anaesthesia for patients with mitochondrial myopathy.* Anaesthesia, 1989. **44**(12): p. 975-7.

70.	Casta, A., et al., *Perioperative white matter degeneration and death in a patient with a defect in mitochondrial oxidative phosphorylation.* Anesthesiology, 1997. **87**(2): p. 420-5.

71.	Gilmore, D.A. and J. Mayhew, *Anesthesia in a child with pyruvate dehydrogenase deficiency: a case report.* AANA J, 2008. **76**(6): p. 432-3.

72.	Grattan-Smith, P.J., et al., *Acute respiratory failure precipitated by general anesthesia in Leigh's syndrome.* J Child Neurol, 1990. **5**(2): p. 137-41.

73.	Gurrieri, C., et al., *Anesthetic considerations in mitochondrial encephalomyopathy, lactic acidosis, and stroke-like episodes syndrome: a case series.* Can J Anaesth, 2011. **58**(8): p. 751-63.

74.	Hashimoto, K., et al., *[Anesthetic management of a patient with mitochondrial encephalomyopathy for renal transplantation].* Masui, 2009. **58**(5): p. 629-32.

75.	Hess, A.S., et al., *Volatile anesthesia for a pediatric patient with very long-chain acyl-coenzyme A dehydrogenase deficiency: A case report.* Paediatr Anaesth, 2018. **28**(3): p. 296-297.

76.	Ishiguro, M., et al., *[Prolonged respiratory depression after fentanyl administration in a patient with mitochondrial encephalomyopathy].* Masui, 2006. **55**(1): p. 73-5.

77.	Izquierdo Villarroya, B., et al., *[General anesthesia in a pregnant patient with Friedreich's ataxia].* Rev Esp Anestesiol Reanim, 2001. **48**(3): p. 156.

78.	Jacobs, T.F., et al., *Anaesthesia for a patient with Leigh's syndrome undergoing surgery for scoliosis.* Acta Anaesthesiol Belg, 2004. **55**(1): p. 57-9.

79.	Keyes, M.A., B.V. Van de Wiele, and S.W. Stead, *Mitochondrial myopathies: an unusual cause of hypotonia in infants and children.* Paediatr Anaesth, 1996. **6**(4): p. 329-35.

80.	Kitoh, T., et al., *Anesthetic management for a patient with Kearns-Sayre syndrome.* Anesth Analg, 1995. **80**(6): p. 1240-2.

81.	Kocamanoglu, I.S. and E. Sarihasan, *Anesthetic management of a pediatric patient with leigh syndrome.* Braz J Anesthesiol, 2013. **63**(2): p. 220-2.

82.	Lauwers, M.H., C. Van Lersberghe, and F. Camu, *Inhalation anaesthesia and the Kearns-Sayre syndrome.* Anaesthesia, 1994. **49**(10): p. 876-8.

83.	Morrison, S., et al., *Anesthetic management of uncorrected tetralogy of fallot and mitochondrial disorder: A role for dexmedetomidine.* Paediatr Anaesth, 2019. **29**(5): p. 539-540.

84.	Mouloudi, H., C. Katsanoulas, and G. Frantzeskos, *Requirements for muscle relaxation in Friedreich's ataxia.* Anaesthesia, 1998. **53**(2): p. 177-80.

85.	Peixoto, C., et al., *Perioperative anesthetic management of very long-chain acyl-coenzyme a dehydrogenase deficiency.* Rev Esp Anestesiol Reanim (Engl Ed), 2023. **70**(4): p. 231-234.

86.	Perilli, V., et al., *[Use of atracurium in patients with muscular diseases. 2 cases of distal and mitochondrial myopathy].* Minerva Anestesiol, 1991. **57**(7-8): p. 441-4.

87.	Serhat Kocamanoglu, I. and E. Sarihasan, *Anesthetic management of a pediatric patient with Leigh syndrome.* Braz J Anesthesiol, 2013. **63**(2): p. 220-2.

88.	To, L.K., et al., *Personalized pediatric ophthalmology: a case report.* J AAPOS, 2019. **23**(4): p. 234-236.

89.	Torres Lopez, A., et al., *[Anesthetic management in a case of Friedreich's ataxia].* Rev Esp Anestesiol Reanim, 1989. **36**(5): p. 286-7.

90.	Vilela, H., et al., *Anesthetic management of a patient with MERRF syndrome.* Paediatr Anaesth, 2005. **15**(1): p. 77-9.

91.	Yamamoto, T., et al., *Anesthetic Management in Mitochondrial Encephalomyopathy: A Case Report.* Anesth Prog, 2017. **64**(4): p. 235-239.

92.	Che, L., et al., *Intraoperative management during liver transplantation in the child with mitochondrial depletion syndrome: A case report.* Int J Surg Case Rep, 2024. **116**: p. 109432.

93.	Chow, S.Y. and K.L. Woon, *General anesthesia for adults with mitochondrial myopathy.* A A Case Rep, 2015. **4**(5): p. 52-7.

94.	Cooper, M.A. and R. Fox, *Anesthesia for corrective spinal surgery in a patient with Leigh's disease.* Anesth Analg, 2003. **97**(5): p. 1539-1541.

95.	Fadrique-Fuentes, A., et al., *Anesthesia outside the Operating Room in a Patient with Mitochondrial Disease.* Case Rep Anesthesiol, 2020. **2020**: p. 7902820.

96.	Humeidan, M.L., J. Dalia, and W.D. Traetow, *Anesthetic considerations for renal transplant surgery in patients with mitochondrial myopathy, encephalopathy, lactic acidosis, and stroke-like episodes syndrome: a case report.* J Clin Anesth, 2016. **34**: p. 344-7.

97.	Kitaura, A., et al., *Nine-Second Cardiac Arrest in a Patient With Anti-mitochondrial Antibody-Positive Myopathy Under General Anesthesia.* Cureus, 2023. **15**(4): p. e37436.

98.	Manickam, A., et al., *Balancing Anesthesia in a Child With Mitochondrial Disease: A Case Report.* Cureus, 2024. **16**(10): p. e70756.

99.	Miyamoto, Y., et al., *Perioperative considerations in adult mitochondrial disease: A case series and a review of 111 cases.* Mitochondrion, 2016. **26**: p. 26-32.

100.	Sibulo, L., et al., *Anesthetic Management of a Child With Propionic Acidemia Complicated by Bacteremia and Severe Acute Respiratory Syndrome Coronavirus 2.* J Med Cases, 2021. **12**(4): p. 152-156.

101.	Tsiotou, A.G., et al., *The child with glutaric aciduria type I: anesthetic and perioperative management.* J Anesth, 2011. **25**(2): p. 301-4.

102.	Valent, A., et al., *Peri-operative management of an adult with POLG-related mitochondrial disease.* Anaesth Rep, 2022. **10**(1): p. e12159.

103.	Valent, A., et al., *Perioperative Management of an Adult Patient With Myoclonic Epilepsy With Ragged Red Fibers Syndrome: A Case Report.* A A Pract, 2021. **15**(2): p. e01401.

104.	Warncke, K.A. and J.M. Marshall, *A Patient With Kearns Sayre Syndrome and Charcot-Marie-Tooth for Supraventricular Tachycardia Ablation: A Case Report.* A A Pract, 2021. **15**(6): p. e01488.

105.	James, R.H., *Induction agent sensitivity and ophthalmoplegia plus.* Anaesthesia, 1986. **41**(2): p. 216.

106.	Melonio, C.E.C., et al., *Anesthesia for bariatric surgery in patient with mitochondrial myopathy - case report.* Braz J Anesthesiol, 2021. **71**(1): p. 87-89.

107.	Mehta, S.D., et al., *Endotracheal Intubation Using Alfentanil in a Pediatric Patient with a Mitochondrial Myopathy and Gastroparesis.* J Pediatr Genet, 2021. **10**(1): p. 53-56.

108.	Nguyen, T., et al., *Anesthetic Management for a Child With a Newly Identified Mitochondrial Disease SLC25A46 Mutation: A Case Report.* Cureus, 2023. **15**(10): p. e47076.

109.	Salehpoor, M.S., et al., *Anesthetic Management of a Patient With Mitochondrial Encephalomyopathy, Lactic Acidosis, and Stroke-Like Episodes Syndrome During Extensive Spinal Surgery With Both Motor Evoked Potentials and Somatosensory Evoked Potentials: A Case Report.* Cureus, 2023. **15**(10): p. e47198.

110.	Huercio, I., et al., *Anaesthesia for orphan disease: combined spinal-epidural anaesthesia in a patient with Friedreich's ataxia.* Eur J Anaesthesiol, 2014. **31**(6): p. 340-1.

111.	Lim, W.Y., et al., *Perioperative considerations of pyruvate dehydrogenase complex deficiency: a case report of two consecutive anesthesia.* Anesth Pain Med (Seoul), 2023. **18**(3): p. 270-274.

112.	Teng, W.N., et al., *Anesthetic management of comprehensive dental restoration in a child with glutaric aciduria type 1 using volatile sevoflurane.* J Chin Med Assoc, 2014. **77**(10): p. 548-51.

113.	Bird, T.M. and L. Strunin, *Hypotensive anesthesia for a patient with Friedreich's ataxia and cardiomyopathy.* Anesthesiology, 1984. **60**(4): p. 377-80.

114.	Dierdorf, S.F. and W.L. McNiece, *Anaesthesia and pyruvate dehydrogenase deficiency.* Can Anaesth Soc J, 1983. **30**(4): p. 413-6.

115.	Pivalizza, E.G., K.J. Ando, and M.S. Sweeney, *Kearns-Sayre syndrome and cardiac anesthesia.* J Cardiothorac Vasc Anesth, 1995. **9**(2): p. 189-91.

116.	Campbell, A.M. and G.A. Finley, *Anaesthesia for a patient with Friedreich's ataxia and cardiomyopathy.* Can J Anaesth, 1989. **36**(1): p. 89-93.

117.	James, R.H., *Thiopentone and ophthalmoplegia plus.* Anaesthesia, 1985. **40**(1): p. 88.

118.	Ward, D.S., *Anesthesia for a child with Leigh's syndrome.* Anesthesiology, 1981. **55**(1): p. 80-1.

119.	Kishida, T., et al., *Successful Perioperative Management of Cochlear Implantation in a Patient With Mitochondrial Encephalopathy, Lactic Acidosis, and Stroke-Like Episodes (MELAS).* Cureus, 2022. **14**(8): p. e27761.

120.	Bell, C.F., J.M. Kelly, and R.S. Jones, *Anaesthesia for Friedreich's ataxia. Case report and review of the literature.* Anaesthesia, 1986. **41**(3): p. 296-301.

121.	Haas, A. and F. Wappler, *[Characteristics of anesthesia in patients with MELAS syndrome: Case report of anesthesia in video-assisted thoracoscopy].* Anaesthesist, 2015. **64**(10): p. 747-53.

122.	Maeda, Y., et al., *[Perioperative administration of bicarbonated solution to a patient with mitochondrial encephalomyopathy].* Masui, 2001. **50**(3): p. 299-303.
